# Enhanced clay formation key in sustaining the Middle Eocene Climatic Optimum

**DOI:** 10.1038/s41561-023-01234-y

**Published:** 2023-07-31

**Authors:** Alexander J. Krause, Appy Sluijs, Robin van der Ploeg, Timothy M. Lenton, Philip A. E. Pogge von Strandmann

**Affiliations:** 1 University College London, Earth Sciences, London, UK; 2 Department of Earth Sciences, Faculty of Geosciences, Utrecht University, Utrecht, The Netherlands; 3 Shell Global Solutions International B.V., Amsterdam, The Netherlands; 4 Global Systems Institute, University of Exeter, Exeter, UK; 5 Institute of Geosciences, Johannes Gutenberg University, Mainz, Germany

**Keywords:** Carbon cycle, Palaeoclimate, Element cycles, Geochemistry, Palaeoceanography

## Abstract

The Middle Eocene Climatic Optimum (around 40 million years ago) was a roughly 400,000-year-long global warming phase associated with an increase in atmospheric CO_2_ concentrations and deep-ocean acidification that interrupted the Eocene’s long-term cooling trend. The unusually long duration, compared with early Eocene global warming phases, is puzzling as temperature-dependent silicate weathering should have provided a negative feedback, drawing down CO_2_ over this timescale. Here we investigate silicate weathering during this climate warming event by measuring lithium isotope ratios (reported as δ^7^Li), which are a tracer for silicate weathering processes, from a suite of open-ocean carbonate-rich sediments. We find a positive δ^7^Li excursion—the only one identified for a warming event so far —of ~3‰. Box model simulations support this signal to reflect a global shift from congruent weathering, with secondary mineral dissolution, to incongruent weathering, with secondary mineral formation. We surmise that, before the climatic optimum, there was considerable soil shielding of the continents. An increase in continental volcanism initiated the warming event, but it was sustained by an increase in clay formation, which sequestered carbonate-forming cations, short-circuiting the carbonate–silicate cycle. Clay mineral dynamics may play an important role in the carbon cycle for climatic events occurring over intermediate (i.e., 100,000 year) timeframes.

## Abstract (web summary)

The long duration of the Middle Eocene Climatic Optimum, compared with other transient Eocene warming events, can be explained by an increase in clays forming from the weathering of silicate minerals, according to lithium isotope records of marine carbonates.

## Main

During the course of the Cenozoic Earth’s surface temperatures have exhibited a long-term reduction^1,2^. However, amidst the backdrop of this general cooling trend, the Earth experienced a number of warming phases on million year timescales, such as the Early Eocene Climatic Optimum^3^. Global warming events on timescales of tens of thousands of years (for example, the Palaeocene–Eocene Thermal Maximum or PETM) were accompanied by a massive input of ^13^C-depleted carbon and deep-sea carbonate dissolution due to ocean acidification, leading to a shoaling of the carbonate compensation depth (CCD)^4–6^. Carbon cycle dynamics across such long- and short-term warming events are relatively well understood^7,8^. However, a warming phase on an intermediate timescale (i.e. 100,000’s of years)—termed the Middle Eocene Climatic Optimum (MECO)—around 40 million years ago (Ma) ^9–11^ has raised interest because it may be inconsistent with the current model of climate–carbon cycle interactions^12^. Although the MECO exhibits similar characteristics to the hyperthermal events of the early Eocene, such as increases in atmospheric CO_2_ ($p_{{\mathrm{CO}}_{2}}$) and temperature, perturbation of the hydrological cycle^13^ and temporary shoaling of the CCD^10,11,14^, its protracted nature (which has been estimated to last between 270 and 500 thousand years (kyr)^9,12,15^) appears to suggest that different dynamics were in operation^12,16^. Over geologic timescales, the chemical weathering of silicate rocks acts as a negative feedback on rising $p_{{\mathrm{CO}}_{2}}$ and temperature levels, thus regulating the climate^7^. Across the MECO, silicate weathering should have kept pace with increases in $p_{{\mathrm{CO}}_{2}}$ and temperature, leading to an enhanced supply of metal cations and alkalinity to the oceans, with a consequent increase in carbonate burial and a reduction in $p_{{\mathrm{CO}}_{2}}$ levels^12^. While other greenhouse warming events were also characterized by transient ocean acidification and shoaling of the CCD (and the lysocline, that is, the depth at which calcite dissolution increases dramatically), the timing from the onset of these events to peak acidification was much more rapid than for the MECO^17^. Thus, the MECO currently stands out as a unique event in the Cenozoic^10,12,18^.

An increase in CO_2_ input to the atmosphere from enhanced volcanic degassing is one hypothesized trigger for the MECO^9^. Conventional carbon cycle theory predicts that, on the timescale of the MECO, injections of volcanic CO_2_ should lead to a deepening of the CCD, since the additional CO_2_ should be converted to alkalinity via continental weathering. However, the available data suggest that the opposite occurred^12^. Osmium (Os) isotope records, combined with carbon cycle box modelling, suggest that a diminished silicate weathering feedback operated during the middle Eocene, enabling the accumulation of volcanic CO_2_ in the exogenic carbon pool^16^. Indeed, a substantial proportion of the present-day Earth has low weathering rates due to soil shielding or aridity^19^. Here we aim to test this hypothesis further by assessing silicate weathering dynamics during the MECO using lithium isotope ratios (δ^7^Li) in marine sediments, followed by model simulations using a newly developed biogeochemical box model.

The dissolution of primary rock shows no lithium isotopic fractionation, whereas the formation of secondary minerals (for example, clays) preferentially take up the light ^6^Li isotope, leaving residual surface waters enriched with the heavier ^7^Li isotope^20–23^. Thus, the δ^7^Li composition of rivers can provide information on the weathering congruency—the ratio of primary rock dissolution versus secondary mineral formation—at that particular time. The importance of clay formation lies not only in its cation-exchange capacity but also in its ability to aid the preservation of buried organic matter, since both organic matter and clays can exhibit a negative surface charge that attracts Mg^2+^ and Ca^2+^, leading to their retention in soils as well as changes in their supply in the dissolved load to the oceans^24–26^. Here we show that this balance between primary mineral dissolution and secondary clay formation is critical for resolving the MECO conundrum.

## Middle Eocene lithium isotope ratios

**Fig. 1:**
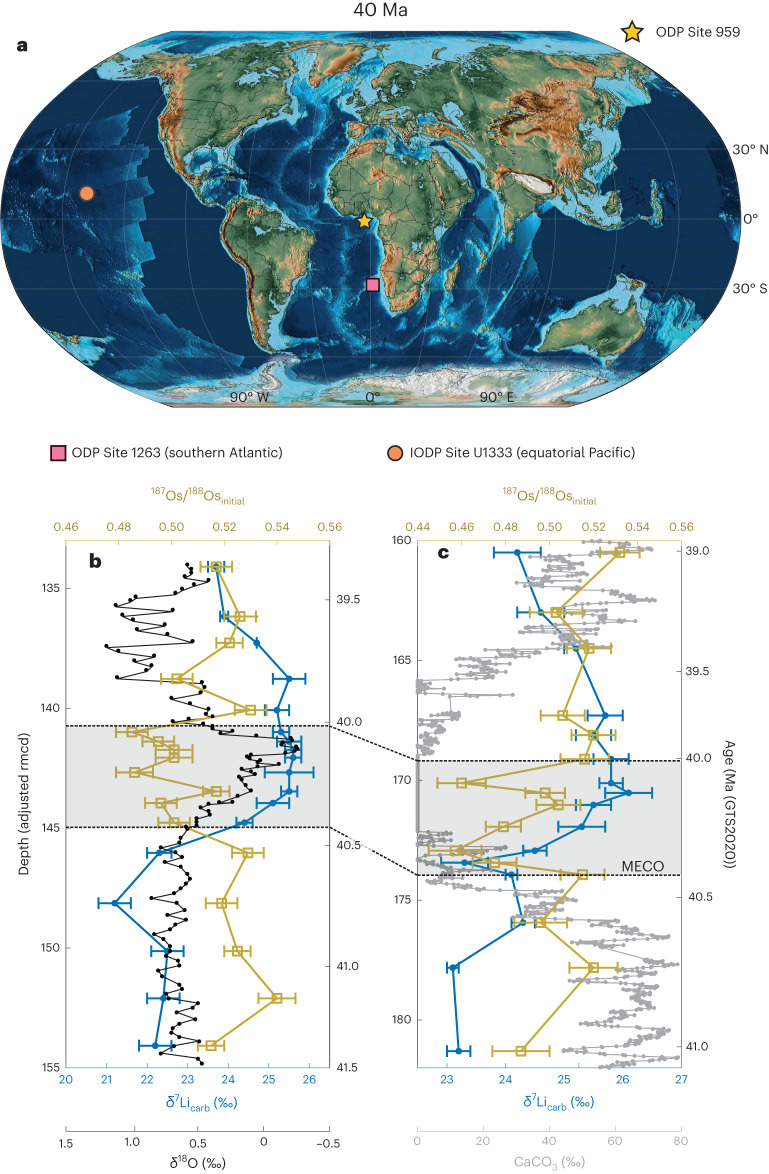
Lithium and osmium isotope records from (I)ODP Sites 1263 and U1333. **a**, Sample site locations and the possible palaeogeography at that time^61^. The general trend of the middle Eocene to the middle Oligocene was one of decreasing flooded continental area^42,62–64^. **b**,**c**, Lithium and osmium isotope records for Site 1263 (**b**) and Site U1333 (**c**), where the filled blue circles represent the mean of each sample (*n* = 3) and the error bars represent the ±2*σ* precision on each δ^7^Li analysis; the ^187^Os/^188^Os data are from ref. ^16^. The grey band represents the estimated duration of the MECO based on oxygen isotope (δ^18^O) data, from a number of ODP sites, tied to an age model framework based on ODP Site 702 (ref. ^18^). Other data previously used^16^ to distinguish the MECO (that is, δ^18^O (ref. ^10^) and CaCO_3_ (ref. ^65^)) are also plotted. All data are plotted against both the adjusted revised metres composite depth (rmcd) and age (GTS2020). Uneven temporal spacing is due to variations in sedimentation rates.

We investigate the response of silicate weathering and clay formation during the MECO by obtaining δ^7^Li data from the same carbonate-rich sediments deposited in pelagic settings that were previously investigated for osmium isotope ratios (^187^Os/^188^Os)^16^, which are together representative of the global ocean. These include the Ocean Drilling Program (ODP) Site 1263 in the South Atlantic (Fig. 1a,b), the Integrated Ocean Drilling Program (IODP) Site U1333 in the equatorial Pacific (Fig. 1a,c) and ODP Site 959 in the equatorial Atlantic (Fig. 1a and Supplementary Fig. 1). We updated existing age models^16^ to convert the core depth to age (based on the 2020 Geologic Time Scale (GTS2020)^27^ and plotted the data for Sites 1263 and U1333 against both depth and age). While Site 959 exhibits the same secular trend for both the lithium and osmium isotopes as Sites 1263 and U1333, indicating that it is recording global changes, its sediment lithology is somewhat anomalous, mainly porcellanite, and the absolute δ^7^Li values are markedly different; thus, we do not include this site in further discussions (see Supplementary Information for more details).

**Fig. 2:**
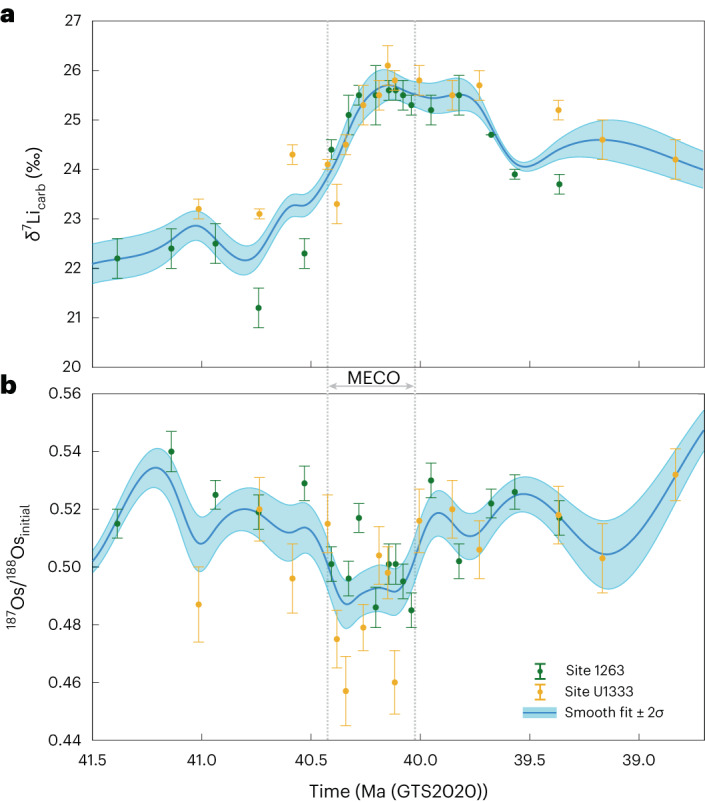
The general trend of the lithium and osmium isotope records across the MECO. **a**,**b**, The data from Sites 1263 and U1333 have been combined (as with Fig. 1, the filled symbols represent the mean ± 2*σ*) and smoothing spline fits (mean ± 2*σ*) have been applied for δ^7^Li_carb_ (**a**) and ^187^Os/^188^Os_initial_ (**b**)^16^. The grey dotted lines indicate the length of the MECO (~40.425 to ~40.023 Ma) based on the δ^18^O data from a number of ODP sites tied to an age model framework based on ODP Site 702^18^. The data are plotted against time (GTS2020).

Both Sites 1263 and U1333 exhibit a positive lithium isotope excursion (LIE) of ~3–3.5‰ across the MECO, with Site 1263 increasing from a pre-MECO value of ~22.1‰ to a peak of 25.6‰ during the event, and U1333 moving from ~23.1‰ to 26.1‰. We combined the isotopic data from Sites 1263 and U1333 and then applied a smoothing spline to obtain a general trend for our timeframe of interest for modelling purposes (41.5–39.0 Ma; Fig. 2 and Methods). Based on an average fractionation factor between seawater and carbonate (Δ^7^Li_sw-carb_) of 4‰ (2–6‰ full range^28–31^), we surmise that, before the MECO, the δ^7^Li of seawater (δ^7^Li_sw_), based on the smoothed average of the two sites, was ~26.1–27.8‰ (full range 24.1–29.8‰), which is in line with previous estimates^20^, and rose to a peak of ~29.7‰ (27.7–31.7‰) between 40.18–40.10 Ma. Seawater pH declined during the MECO^18^, and although pH has been shown to affect δ^7^Li fractionation somewhat^32^, the change in pH would have resulted in only a minor change in δ^7^Li fractionation, suggesting that an impact on our dataset is unlikely. Furthermore, the absolute effect of pH on δ^7^Li fractionation is still contested^33^.

Given the ~1 Myr residence time of lithium in the present ocean^20^, the shift in δ^7^Li within a few 100 kyrs indicates, geologically speaking, a fairly rapid and strong change in the Earth system conditions. Consistent with the long residence time is the observation that, by 39 Ma, the δ^7^Li of carbonates (δ^7^Li_carb_) is still higher than before the MECO. The beginning of the positive LIE appears to occur at approximately the same time as the negative osmium isotope excursion (OsIE), with both isotope systems exhibiting changes at ~40.5 Ma just before the start of the MECO, while the seawater ^187^Os/^188^Os_initial_ (reflecting the osmium isotope ratio of marine sediments at the time of their deposition) recovers to pre-MECO values at, or just after, 40 Ma. The faster recovery time of the OsIE may be explained by the shorter residence time of osmium in the ocean (5–54 kyr)^34^. Furthermore, the opposite signs of the LIE and OsIE are contrary to previous work, where both isotope systems change in tandem; whereas a positive LIE has hitherto been associated with cooling events, warming events have produced negative LIEs^29,35,36^. A negative OsIE could be explained by an increased flux of high-temperature hydrothermal fluids, which would supply unradiogenic osmium to the ocean^16,34^. However, this would also deliver isotopically light δ^7^Li (refs. ^20,37^), causing a negative LIE, in contrast to our results from all three (I)ODP sites.

## Modelling Earth system changes during the MECO

To assess the background Earth system state before the MECO and the changes that occurred during the event, we explored a number of scenarios using our newly developed CARLIOS biogeochemical box model (see Methods and Supplementary Information for further details about the model). For all scenarios we attempted to approximate the $p_{{\mathrm{CO}}_{2}}$ and temperature proxy data (see Supplementary Information for details on the proxies) as well as our δ^7^Li and ^187^Os/^188^Os data, and to reproduce some general characteristics of the MECO, such as a decrease in the surface ocean pH and a shoaling of the lysocline (Table 1).

**Table 1:** CARLIOS model simulations assessed against key MECO metrics

Scenarios	$p_{{\mathrm{CO}}_{2}}$	Surface pH	δ^7^Li	^187^Os/^188^Os	Lysocline	Result
(1) CO_2_ input to the atmosphere	↑	↓	No match	No match	↓	Failure
(2) Big increase in normalized uplift	↓	↑	Good match	No match	↑	Failure
(3) Increase in reverse weathering	↑	↓	Reasonable match	No match	=	Failure
(4) Decrease in marine organic carbon burial	↑	Small ↓	No match	No match	↓	Failure
(5) Shift more carbonate carbon burial to shelves	=	=	No match	No match	↓	Failure
(6) CO_2_ input plus a big increase in uplift	↑	↑	Good match	No match	Small ↑	Failure
(7) CO_2_ input plus a small increase in uplift, a change in the erosion-to-silicate-weathering ratio and a pulse of unradiogenic osmium	↑	Small ↓	Good match	Good match	↓	Failure
(8) As Scenario 7 plus a decline in both the [Mg]_sw_ and the carbonate land area	↑	↓	Good match	Good match	↑	Success

**Fig. 3:**
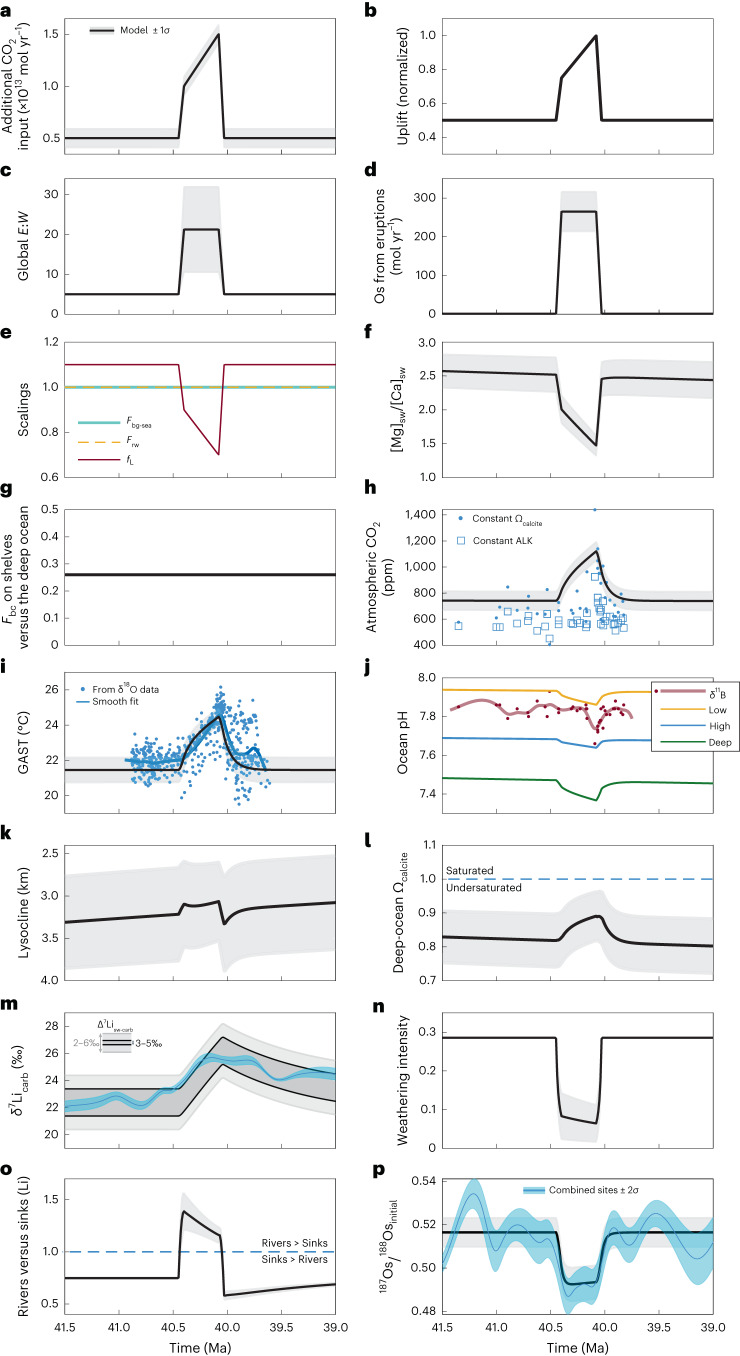
Key parameter changes to the CARLIOS model and its results for Scenario 8. **a**–**g**, Parameter changes for: an injection of CO_2_ to the atmosphere (**a**), a change in the normalized uplift (**b**), a change to the global erosion-to-silicate-weathering ratio (*E*:*W*) (**c**), a pulsed continental eruption of unradiogenic osmium which is then subaerially weathered (**d**), scalings for the burial of marine organic carbon (F_bg-sea_, blue), reverse weathering (F_rw_, dashed gold) and the carbonate land area (f_L_, red) (**e**), a change to the Mg/Ca ratio in the oceans ([Mg]_sw_/[Ca]_sw_) (**f**) and the factor that apportions carbonate burial (*F*_bc_) to shelf environments, where a lower number indicates more burial on shelves (**g**). **h**–**p**, Key model results for atmospheric $p_{{\mathrm{CO}}_{2}}$ (black) plotted against proxy estimations from boron isotope (δ^11^B) data^18^ (see Supplementary Information) (**h**), global average surface temperature (GAST) plotted against proxy estimations from δ^18^O data (see Supplementary Information) (**i**), ocean pH (for a low-latitude surface (gold), a high-latitude surface (blue) and a deep ocean (green), and where the red dots and line denote individual pH estimations from δ^11^B data^18^ and a smoothed fit, respectively) (**j**), modelled lysocline depth (**k**), deep-ocean Ω_calcite_ saturation (**l**), the modelled mean δ^7^Li_sw_ offset by a fractionation factor (3–5‰, dark grey band; 2–6‰, light grey band) (**m**), the modelled weathering intensity through time (**n**), the contribution of rivers versus the combined sinks to the δ^7^Li_sw_ signature (**o**) and the modelled ^187^Os/^188^Os_initial_ of seawater (**p**). In all panels (apart from **e**, **j** and **m**) the black line is the model average and the grey band denotes ±1*σ*. In **m** and **p**, the blue line and band are from Fig. 2. ALK, ocean alkalinity.

**Extended Data Fig. 1:**
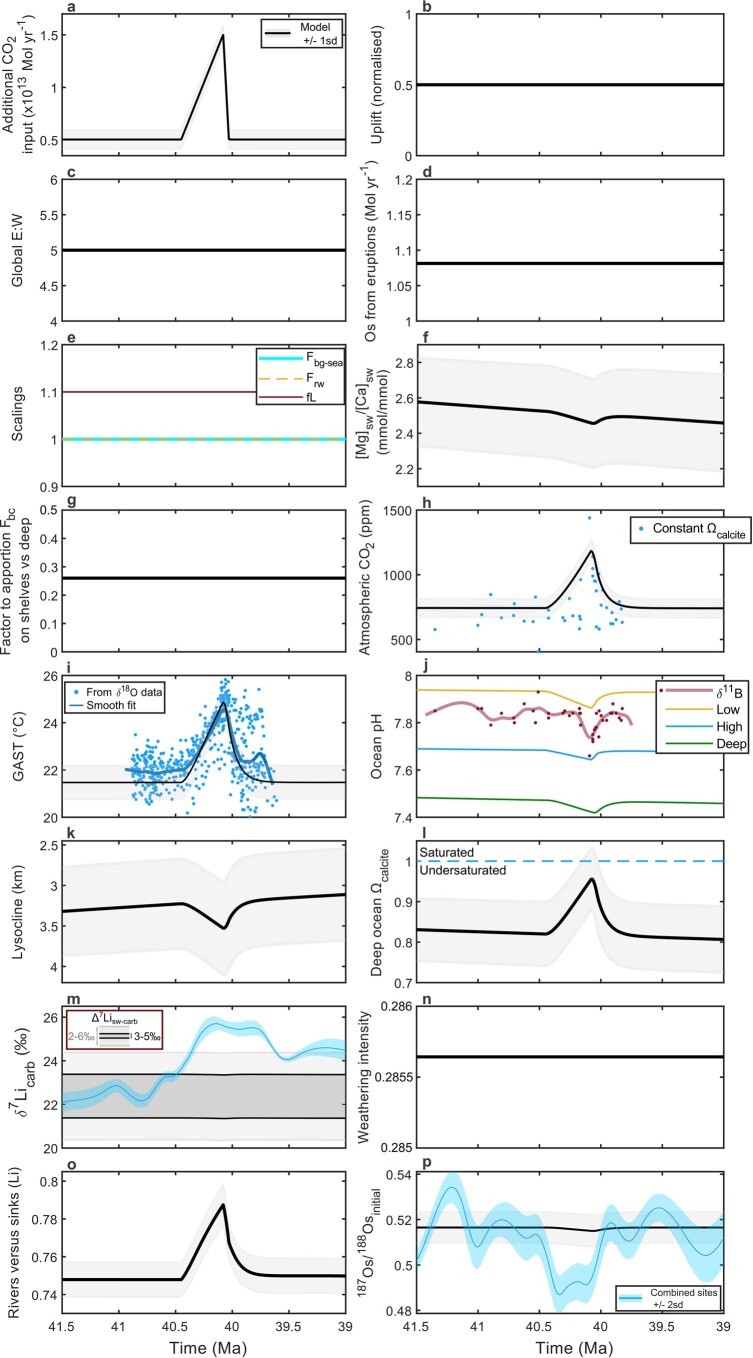
Failed scenario 1, a modelled increase in CO_2_ directly to the atmosphere. Panels **a**–**g** are parameter changes and panels **h**–**p** are key model results as detailed for Main Fig. 3. In all panels (apart from e, j and m) the black line is the model average and the grey band is the ±1σ. In panels m and p the blue line and band are from Main Fig. 2.

**Extended Data Fig. 2:**
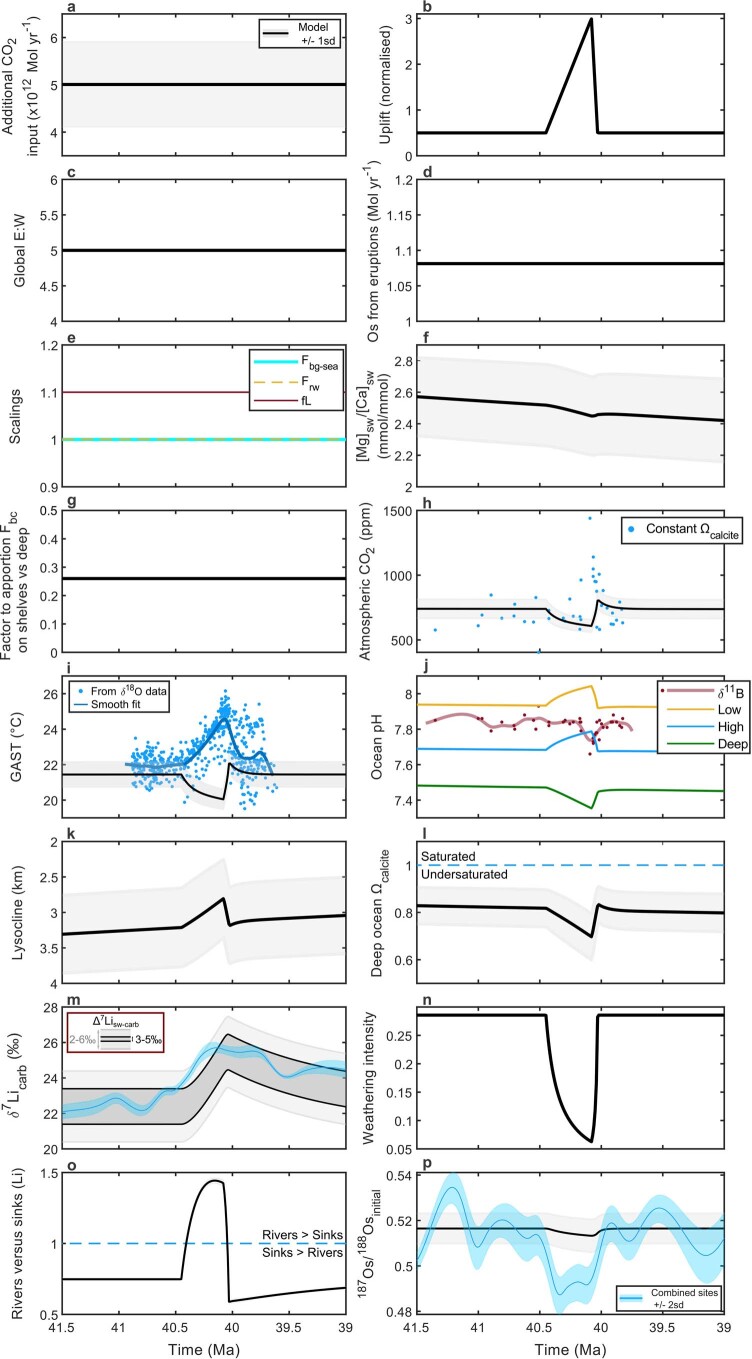
Failed scenario 2, a modelled large increase in uplift (and thus erosion). Panels **a**–**g** are parameter changes and panels **h**–**p** are key model results as detailed for Main Fig. 3. In all panels (apart from e, j and m) the black line is the model average and the grey band is the ±1σ. In panels m and p the blue line and band are from Main Fig. 2.

**Extended Data Fig. 3:**
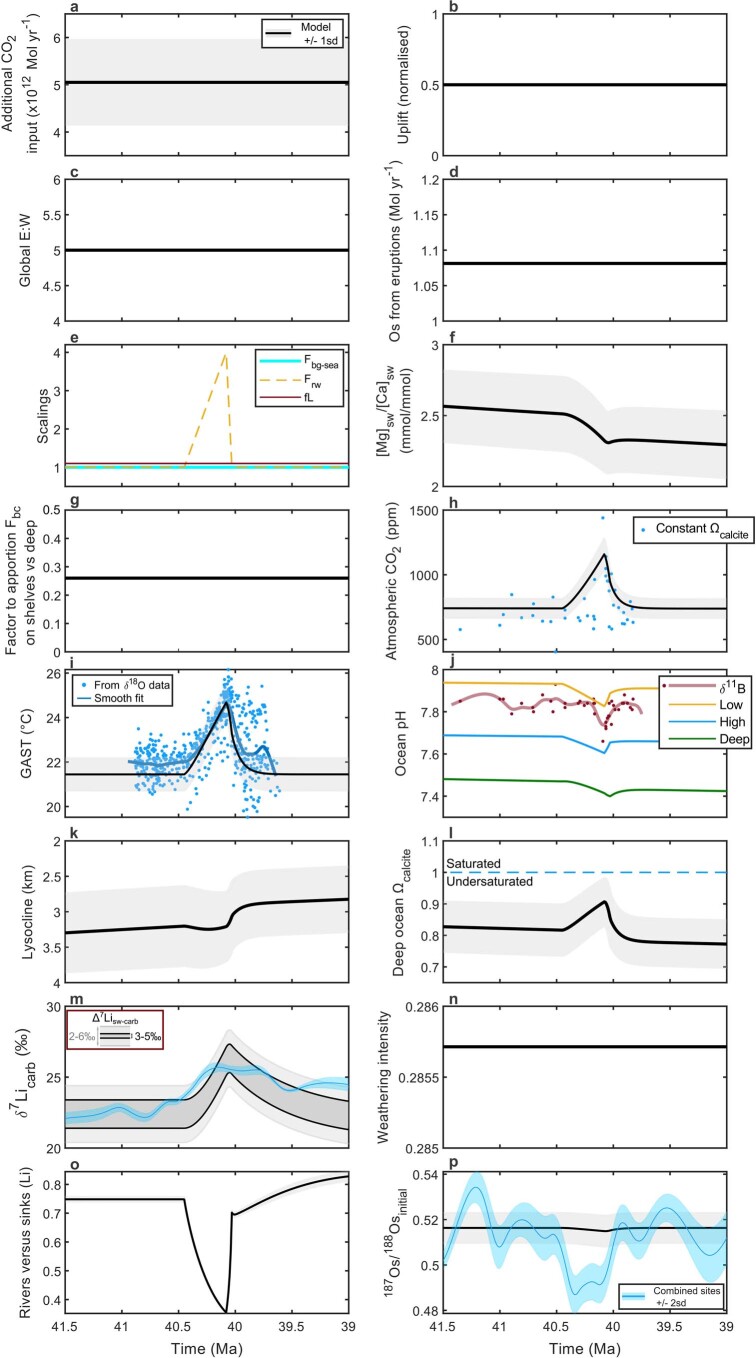
Failed scenario 3, a modelled increase in reverse weathering. Panels **a**–**g** are parameter changes and panels **h**–**p** are key model results as detailed for Main Fig. 3. In all panels (apart from e, j and m) the black line is the model average and the grey band is the ±1σ. In panels m and p the blue line and band are from Main Fig. 2.

**Extended Data Fig. 4:**
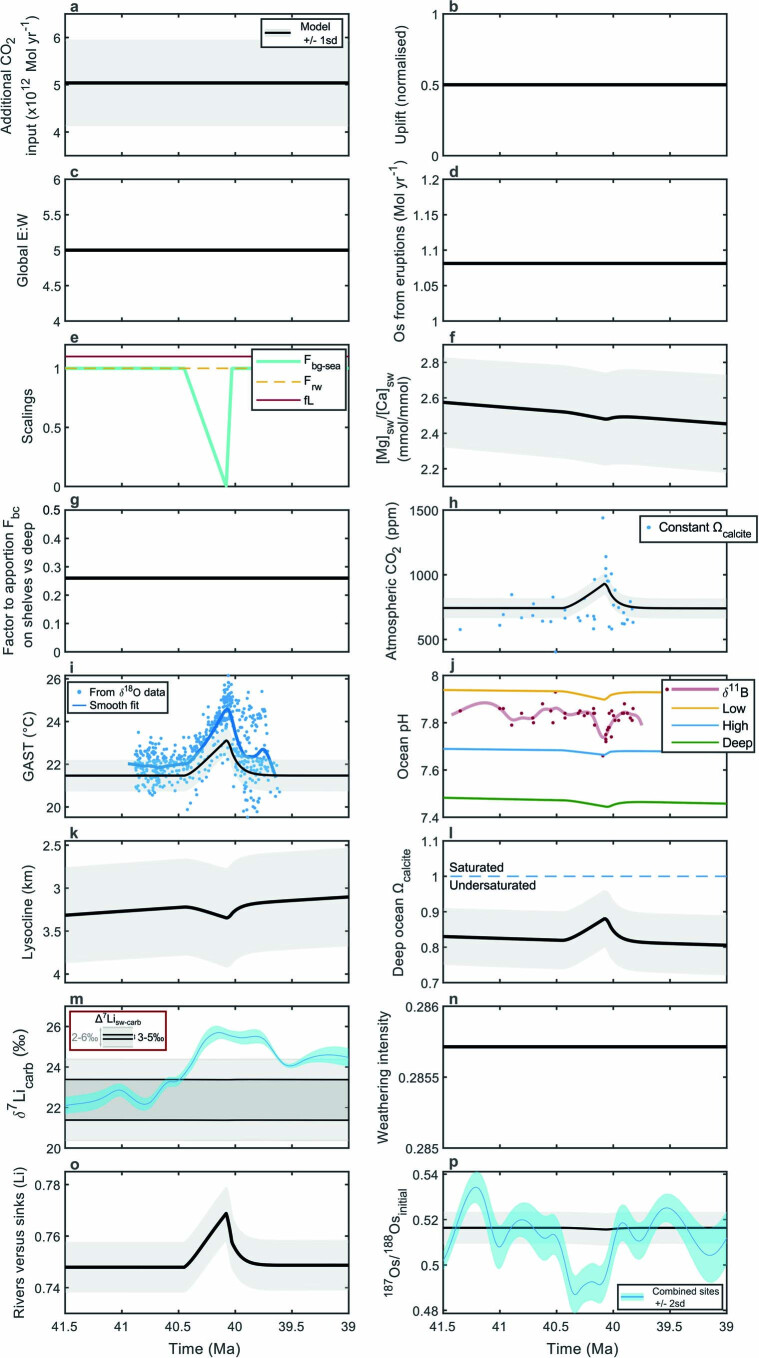
Failed scenario 4, a modelled decrease in marine organic carbon burial. Panels **a**–**g** are parameter changes and panels **h**–**p** are key model results as detailed for Main Fig. 3. In all panels (apart from e, j and m) the black line is the model average and the grey band is the ±1σ. In panels m and p the blue line and band are from Main Fig. 2.

**Extended Data Fig. 5:**
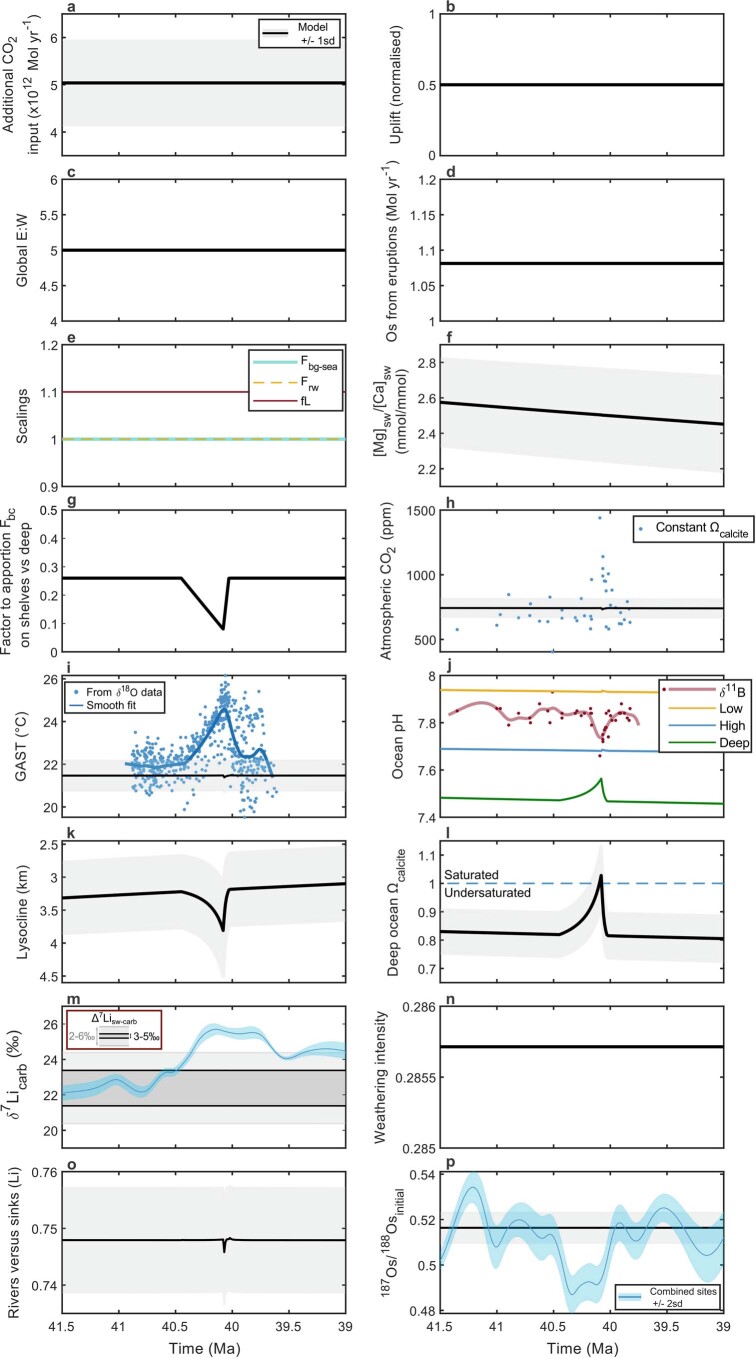
Failed scenario 5, a modelled shift in carbonate burial. Panels **a**–**g** are parameter changes and panels **h**–**p** are key model results as detailed for Main Fig. 3. In all panels (apart from e, j and m) the black line is the model average and the grey band is the ±1σ. In panels m and p the blue line and band are from Main Fig. 2.

**Extended Data Fig. 6:**
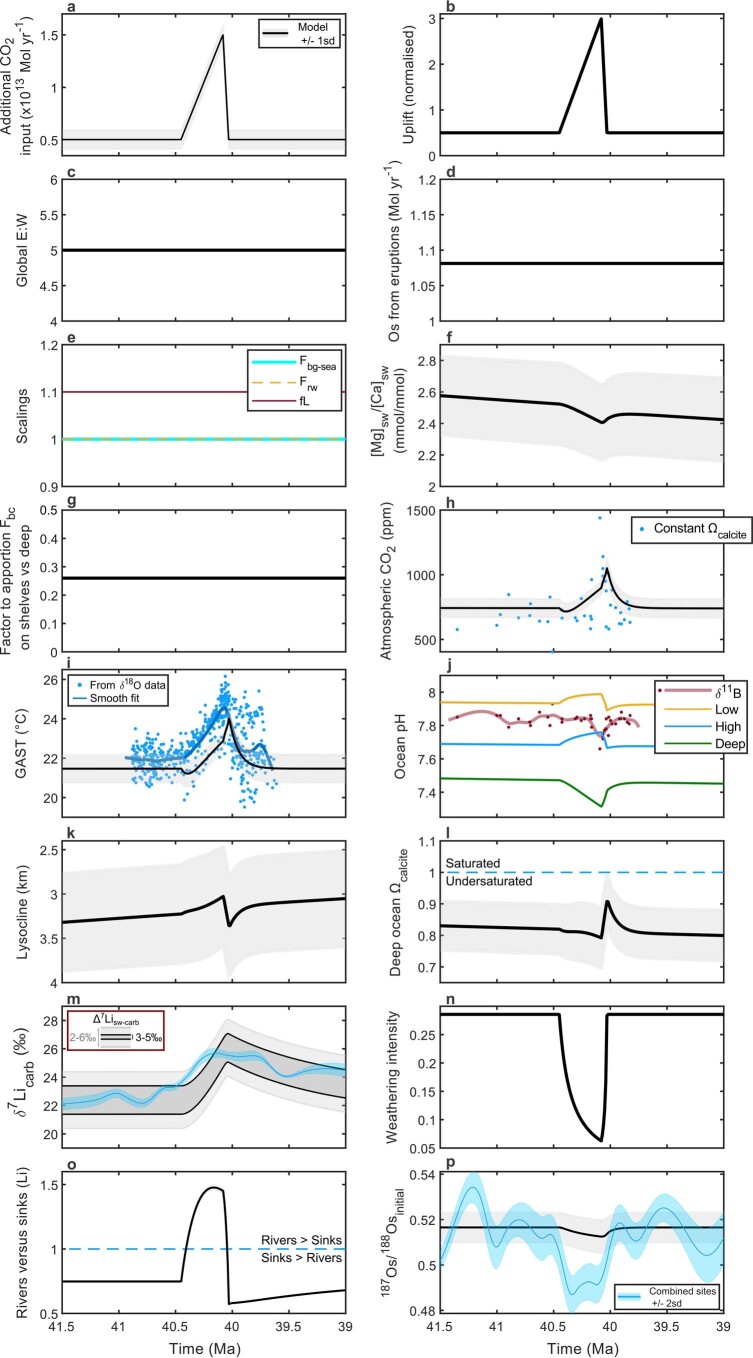
Failed scenario 6, a modelled increase in CO_2_ and a large increase in uplift. Panels **a**–**g** are parameter changes and panels **h**–**p** are key model results as detailed for Main Fig. 3. In all panels (apart from e, j and m) the black line is the model average and the grey band is the ±1σ. In panels m and p the blue line and band are from Main Fig. 2.

**Extended Data Fig. 7:**
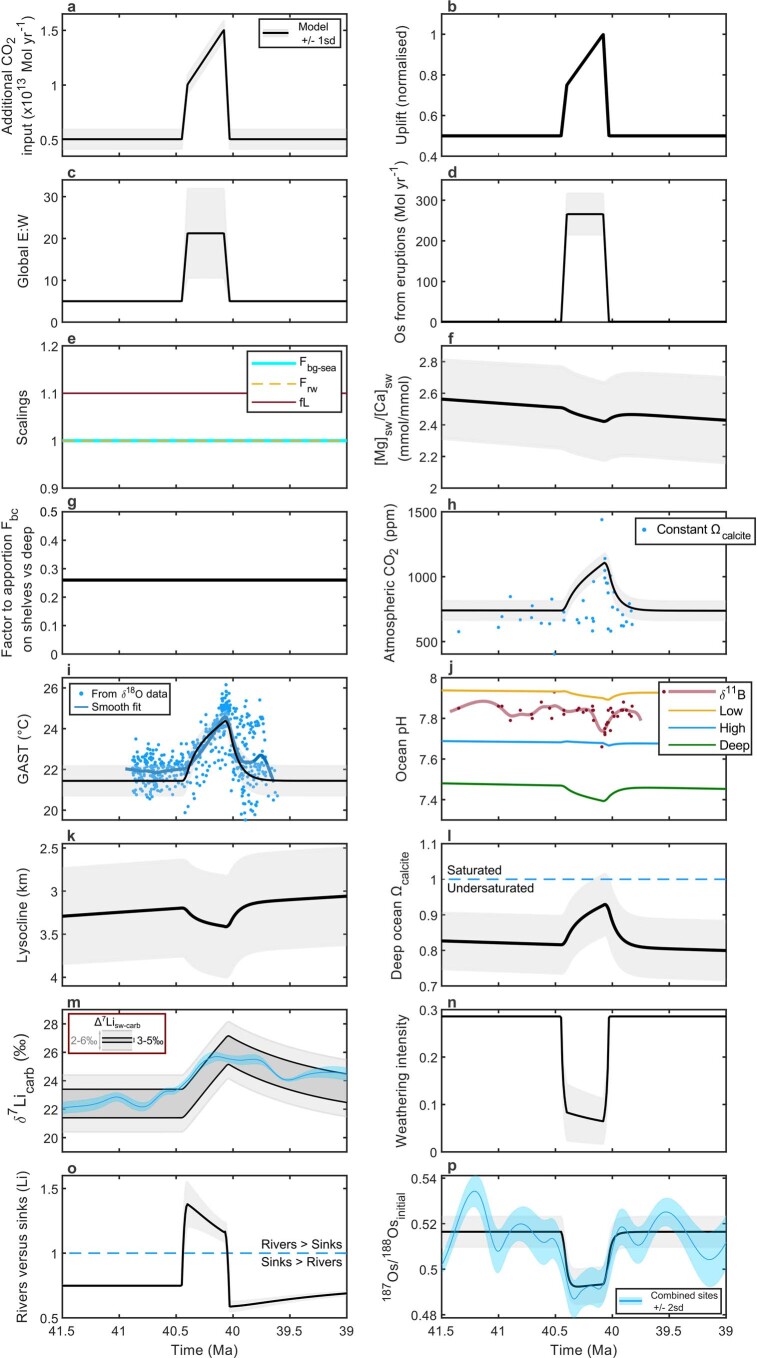
Failed scenario 7, a modelled increase in CO_2_, a moderate increase in uplift, a change in the global erosion to silicate weathering ratio and an increase in mantle-like unradiogenic Os. Panels **a**–**g** are parameter changes and panels **h**–**p** are key model results as detailed for Main Fig. 3. In all panels (apart from e, j and m) the black line is the model average and the grey band is the ±1σ. In panels m and p the blue line and band are from Main Fig. 2.

All scenarios (1–8) in Table 1 can capture one or more of the main MECO characteristics (Fig. 3 and Extended Data Figs. 1–7); however, although Scenarios 1, 3 and 4, for example, produce an increase in the $p_{{\mathrm{CO}}_{2}}$ (and temperature) and a decrease in the ocean pH, none of these scenarios shoal the lysocline, and Scenarios 1 and 4 do not produce a good fit to the δ^7^Li and ^187^Os/^188^Os data. An increase in normalized uplift only (Scenario 2) does raise the lysocline depth and matches the δ^7^Li data, but reduces $p_{{\mathrm{CO}}_{2}}$ while the surface pH increases, contrary to the proxy data. Shifting more of the carbonate burial from deep sediments to the continental shelves (Scenario 5), to reflect platform flooding due to thermal expansion of seawater, barely makes a difference to the background (pre- and post-MECO) results of the model, thus in previous modelling^12^ this parameter change may have made little difference to the results. Combining the parameters of Scenarios 1 and 2, we model an injection of CO_2_ directly to the atmosphere that occurs concurrently with a big increase in uplift (Scenario 6). Although this raises both $p_{{\mathrm{CO}}_{2}}$ and the lysocline, and approximately matches our δ^7^Li data, the pH also increases. None of the changes in Scenarios 1–6 produce a match to the ^187^Os/^188^Os data. For Scenario 7, we again added CO_2_ to the atmosphere, albeit with a different functional form to Scenarios 1 and 6 (compare Extended Data Figs. 6a and 7a), modelled a relatively modest increase in uplift, allowed the global erosion-to-silicate-weathering ratio to vary (this ratio is used to help convert normalized uplift to erosion in tonnes per year), and increased the flux of unradiogenic osmium from continental eruptions (see Supplementary Information for more details about the osmium cycle). Scenario 7 produced results which were a good fit to almost all key aspects of the MECO; however, the lysocline deepened. Thus, for Scenario 8 we used all of the parameters from Scenario 7 but hypothesized that there may have been two additional changes during the MECO: a decline in the concentration of magnesium in seawater ([Mg]_sw_), which affects the Mg/Ca ratio of seawater and the calcite solubility product (see below); and a reduction in the total areal exposure of carbonate rocks to weathering^12^. This scenario (Fig. 3) successfully captured the key aspects of the MECO as listed in Table 1. Although the calcite saturation state (Ω_calcite_) in this scenario does increase slightly, it remains at <1, meaning that carbonate dissolution will still outweigh sedimentation, and hence we use Scenario 8 as our chosen scenario for further discussion.

## A global shift towards enhanced clay formation

Our model scenarios indicate that, as with previous work^12^, no single mechanism in isolation can reproduce the carbon cycle characteristics of the MECO and match the δ^7^Li and ^187^Os/^188^Os data. However, individual scenarios are useful for understanding the various impacts on the Earth system. For example, Scenario 2 indicates that the global weathering intensity (*W*/*D*)—the ratio of chemical weathering *W* to total denudation *D* (which is *W* plus erosion *E*)—decreased across the MECO, implying that there was a shift in the Earth’s weathering regime^30^ from clay dissolution to clay formation, on a global average.

Calcium (Ca) and magnesium cations are attracted to the negatively charged surface layers of some clays and are structural components of others (for example, smectites). With increased terrestrial clay formation as suggested by the δ^7^Li data, it follows that more calcium and magnesium will be retained in soils, leaving a deficit of oceanic calcium and magnesium to form carbonates and close the carbonate–silicate cycle^24,26^. Variable calcium and magnesium concentrations are observed in modern rivers (for example, ref. ^22^), and more work needs to be conducted on the relationship between riverine δ^7^Li and cation concentrations. Many marine clay-formation reactions (reverse weathering) also take up calcium and magnesium, as well as bicarbonate ions, releasing CO_2_ to the oceans^38^. It is currently unknown by how much terrestrial calcium and magnesium retention scales with clay formation, and thus the subsequent impact on atmospheric CO_2_ is equally unknown. It has been shown that magnesium is less mobile than calcium^39^ and is therefore trapped by clays more efficiently; magnesium is also taken up in higher molar ratios during reverse weathering reactions^38^. Thus, clay formation, either on land or in the oceans, will probably impact upon the magnesium cycle and its availability in the oceans for carbonate-forming reactions more than calcium. Although magnesium currently has a long residence time (~13 Myr), [Mg]_sw_ at ~40 Ma was much lower than today (~38.4 mmol compared with ~53 mmol)^40^. The inclusion of this reduction in the Mg/Ca ratio of seawater in our model (Scenario 8) helps to shoal the lysocline (compared with Scenario 7) because it affects the calcite solubility product, which then affects the calcite saturation with subsequent knock-on effects for the carbonate system in the oceans.

**Fig. 4:**
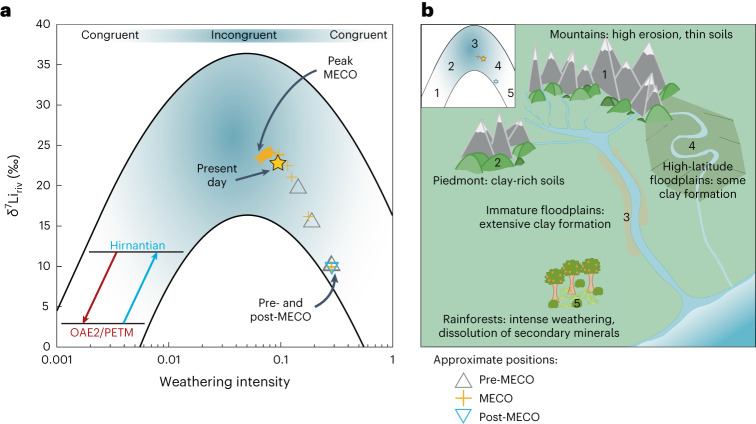
Possible changes to the Earth system as inferred by δ^7^Li data and model results. **a**, The modern-day relationship between *W*/*D* and δ^7^Li_riv_ (the boomerang shape), with which our modelled average *W*/*D* and δ^7^Li_riv_ results from Scenario 8 are compared, as well as those inferred from the modelling of other climatic events (where the *W*/*D* and δ^7^Li_riv_ data decline for Oceanic Anoxic Event 2 (OAE2) and the PETM (red arrow) but increase for the Hirnantian glaciation (blue arrow)); the present-day global average is also shown^21,29,30,35,36^. **b**, The inset shows the pre- and post-MECO conditions (grey and blue triangles near position 4), the peak MECO conditions (yellow cross near position 3) and the present-day conditions (yellow star) and how they correspond to the different surface environments (main image) that affect δ^7^Li_riv_ values^30^.

We plotted the weathering intensity (*W*/*D*) and δ^7^Li of rivers (δ^7^Li_riv_) results for Scenario 8 against the ‘Dellinger boomerang’^21^ (Fig. 4a) to determine more precisely the changes in the global *W*/*D* regime. Before the onset of the MECO, owing to a relatively low background erosion rate^41^, a steadily declining sea level and areal extent of flooded continent^42^, our modelling suggests that the Earth predominately had a mixture of mature floodplains in the high latitudes (Fig. 4a,b, position 4) with areas of tropical weathering (Fig. 4a,b, position 5), although the Earth would still have had mountainous areas with rapid erosion (Fig. 4b, position 1). This may have resulted globally in a net amount of secondary mineral dissolution^30^. Data from the continental interior of North America^43^ and from South America^44^ suggest that warm and wet conditions prevailed at the beginning and end of the MECO. Fully coupled climate model simulations for 38 Ma, validated by field reconstructions, suggest near-surface air temperatures of >20 °C from the 30° to 50° latitudes and >30 °C from the equator to the ±30° latitudes^45^. This would indicate that the background state before (and after) the MECO provided ideal conditions for extensive tropical weathering, although, just as in the present day, there would have been some areas that experienced aridity (for example, refs. ^19,46^). Hence, we postulate there was, globally, more intense weathering, although more climatic studies of the MECO are required to validate this hypothesis.

An increase in CO_2_ input to the ocean-atmosphere from volcanic CO_2_ degassing, as well as from organic carbon oxidation and sulfide oxidation (leading to sulfuric acid weathering of carbonates) due to surface exposure after erosion^47^, would have increased $p_{{\mathrm{CO}}_{2}}$ levels and consequently global surface temperatures. This in turn would probably have resulted in changes to the hydrological cycle and thermal expansion of surficial bodies of water. Several papers have suggested a sea-level rise across the MECO (for example, ref. ^42^) and several recorded transgressive sediment sequences seemingly correspond to the MECO, but the age uncertainties associated with all of these data are greater than the duration of the MECO itself^48^. Nevertheless, an enhanced hydrological cycle^13^ and thermal expansion would have increased both the areal extent of silicate weathering and the water–rock interaction time via the formation of new floodplains^49^. At the same time, thermal expansion may have flooded shelf environments, minimizing the land area available for carbonate weathering but increasing the areal extent of shallow seas from which carbonates could precipitate (for example, ref. ^12^ and references therein), which would have reduced the alkalinity supply and thus carbonate accumulation rates in the deep ocean.

An increase in volcanic activity globally during the MECO would have increased the global erosion rate due not only to a warming-driven increase in rainfall frequency and intensity but also from incision of the underlying bedrock by lava flows, and a combination of the two (increased precipitation and bedrock incision) can manifest itself in lahar events^50–52^. Such an increase in erosion rates would have provided fresh primary minerals to the newly formed floodplains, which in turn provided an ideal environment for weathering and the subsequent formation of secondary minerals (Fig. 4b, position 3), leaving residual riverine waters enriched in the ^7^Li isotope^21,30^.

An increase in secondary mineral formation would have led to more entrainment of Ca^2+^ and Mg^2+^ within these locales, thus reducing the flux of carbonate-forming cations to the oceans. This likely led to a disruption in the carbonate–silicate cycle between the dissolution of terrestrial silicate rocks and the formation of marine carbonates (that is, silicate weathering became less efficient^53^), resulting in a positive feedback loop whereby the retention of Ca^2+^ and Mg^2+^ in the terrestrial realm led to an accumulation of CO_2_ in the atmosphere. Consequently, rates of silicate weathering increased, liberating more Ca^2+^ and Mg^2+^ to be taken up by secondary minerals. The positive feedback loop may only have abated once the supply of primary minerals had declined sufficiently such that clay dissolution once more outweighed clay formation, leading to a net release of Ca^2+^ and Mg^2+^ back to the oceans and potentially to an overshoot of the CCD, although the currently available data are not of sufficient resolution to be able to evaluate this proposition. Alternatively, a short-lived pulse of CO_2_ centred around the MECO ‘peak’, as suggested by the pH decline^18^, may have induced rapid hydrological changes such that the global weathering regime became congruent and the cation flux to the oceans increased considerably. Our model suggests that the global average weathering regime on Earth during the MECO was not dissimilar to that at present (Fig. 4). Assuming that the amount of magnesium and calcium in soils before the MECO is the same as at present (6.10 × 10^14^ t), then by the end of the MECO, assuming a duration of 400 kyr, we calculate that the amount of magnesium and calcium retained in soils would be 6.227 × 10^14^ t, an increase of ~2%, which we believe is a feasible amount of cations stored (see Supplementary Information for derivation). Reverse weathering may also have had an important part to play in terms of reducing the concentration of magnesium and calcium in the oceans while also contributing more CO_2_ to the ocean-atmosphere system^38,54^. Our model suggests that, pre- and post-MECO, the influence of sinks outweighed rivers (rivers/sinks < 1; Fig. 3o), but during the MECO the rivers had a greater control on the δ^7^Li signature of the oceans (rivers/sinks > 1). However, this is not to say that the sinks of lithium declined; indeed, there is evidence for an increase in abyssal smectite precipitation during the MECO^46,55^, but proportionally these sinks were dwarfed by riverine changes.

All previously analysed global warming events, both long-term (OAE2)^29^ and transient (PETM)^36^, exhibit a negative LIE, which is interpreted as a relatively greater increase in erosion rates over weathering rates (causing a decrease in *W*/*D*). This is attributed to an accelerated hydrological cycle, consistent with modern river observations (Fig. 4a)^56^. Hence, the MECO is the only warming event identified so far with a positive LIE. However, the mechanisms underlying changes to the weathering regime remain the same across all warming events analysed so far: the erosion rate increases relative to the weathering rate. The direction of the LIE then depends on the pre-event weathering regime (Fig. 4), which for the MECO is at a vastly higher *W*/*D* than the other hyperthermal events analysed so far.

Our results appear to indicate that silicate weathering continued at pace but was dwarfed by the increase in erosion. Global net secondary mineral formation increased, trapping carbonate-forming cations on the continents, effectively making the link between silicate weathering and $p_{{\mathrm{CO}}_{2}}$ drawdown less efficient. In addition, warmer deep-ocean waters possibly increased the reverse weathering rate, trapping more metal cations and exchanging bicarbonate ions for CO_2_. Consequently, only a modest injection of CO_2_ would have been required to kickstart the MECO, which is in line with other work^18^, and would help explain why there is no global negative δ^13^C excursion associated with the onset of the MECO^12,57^. A spike in CO_2_ degassing during the MECO, as modelled in Scenarios 1, 6, 7 and 8, may explain the rise in $p_{{\mathrm{CO}}_{2}}$ suggested by δ^11^B (ref. ^18^). Once the fresh supply of primary minerals started to decline, the warm climate^14,45^ likely facilitated a return to net clay dissolution, providing the oceans with some Ca^2+^ and Mg^2+^, leading to an increase in carbonate burial^10^. Importantly, a flux of partially dissolved clays to the oceans may have provided microsites for enhanced marine organic carbon burial, whilst also liberating clay-bound PO_4_^3−^ that would have stimulated primary productivity, resulting in an overall decline in $p_{{\mathrm{CO}}_{2}}$ (ref. ^58^). In addition, it has been shown that bacterial metabolic rates are sensitive to temperature changes, with a greater efficiency for remineralizing organic matter at higher temperatures^59,60^. This may have created a positive feedback, which accelerated both MECO warming and post-MECO cooling.

For CO_2_ drawdown to be determined correctly, changes in the silicate dissolution rate must be tempered with changes to the weathering regime. It is the latter that not only changes due to rapid alteration of the climate but is also highly dependent on the pre-perturbation palaeogeography, climate and vegetation regimes. While this is true for all climatic events, it appears that on intermediate timescales, such as those of the MECO, the dynamics of clay minerals may play a particularly important role in the global carbon cycle, effectively disrupting the negative feedback system that regulates $p_{{\mathrm{CO}}_{2}}$ and temperature. Hence, we recommend further investigations of the impact of clay dynamics on intermediate-timescale events in a model with more complex terrestrial and marine carbonate and clay chemistries.

## Methods

### Samples

Carbonate samples were analysed as detailed in previous work^31,36^. Briefly, the samples were leached in 1 M sodium acetate buffered to pH 5 with acetic acid at room temperature. A split (~20%) of the subsequent leachate was analysed via quadrupole inductively coupled plasma mass spectrometry, using matrix-matched calibration standards and the JLs-1 reference material as an accuracy standard, to determine the element/Ca ratios. This is in part to determine ratios such as Al/Ca and Rb/Ca, to ascertain that negligible silicate material has been leached. Cutoffs for the silicate contribution on Al/Ca ratios are suggested as <0.8 mmol mol^−1^ (ref. ^29^) and on Al/(Ca + Mg) as <0.45 mmol mol^−1^ (ref. ^66^). Meanwhile, a cutoff of around <30 μmol mol^−1^ for Rb/Ca has been suggested^67^. All of our samples fit within these parameters (see Supplementary Tables 2 and 3). The remaining 80% of the sample was purified through a two-step cation-exchange method, using 0.2 M HCl as the eluent^68^, and analysed relative to the lithium isotopic reference material IRMM-016 using a Nu Plasma 3 multi collector ICP mass spectrometer (Nu Instruments) at the LoGIC laboratories of University College London. All samples were renormalized to the LSVEC reference material, and the analytical uncertainty was propagated to account for this^69^. Using an Aridus II desolvator, a signal intensity of ~130 pA (~13 V) on ^7^Li for a solution of 5 ng ml^−1^ was achieved (using 10^11^ Ω resistors and an uptake rate of ~100 μl min^−1^, and between 1 and 5 ng lithium was analysed), which is much greater than the background and total procedural blank of 0.01 pA (<1 mV)^36^. The results of several different rock standards analysed using this method have been reported previously^69^, and seawater gives δ^7^Li = 31.18 ± 0.38‰ (2*σ*; *n* = 29). Of particular relevance for low-concentration carbonate samples is that aliquots of seawater (~3 µl) that were purified and then analysed at concentrations of 0.5 ng ml^−1^ (*n* = 6) yield a long-term analytical uncertainty of ±0.4‰ (2*σ*)^36^.

In addition to assessing whether or not our samples had been affected by silicate leaching, we used elemental ratios to evaluate if the samples from Sites 1263 and U1333 had been subject to diagenetic alteration or other forms of contamination (see Supplementary Tables 2 and 3). Both sites show little correlation between the elemental ratios (Mn/Ca, Rb/Ca, Al/Ca) and δ^7^Li, indicating that there is no effect from manganese oxyhydroxides and, as mentioned above, little to no contamination from the leaching of silicates. Although there is a short-lived peak in Mn/Ca during the MECO at Site U1333, there is no clear change in ratios during the event at Site 1263, and no identifiable trends in Rb/Ca across the MECO at either site. Likewise, there is little correlation between Li/Ca and δ^7^Li, and no clear changes in Li/Ca across the MECO. The Li/Ca values are lower than values from the PETM^36^, but are very similar to those measured in the very pure marine carbonates at OAE1a and OAE2^29,70^. Comparing our trace element analyses to recent work on assessing diagenetic affects on δ^7^Li in carbonates^66^ indicates insignificant diagenetic alteration of our samples from Sites 1263 and U1333, and, combined with the similar δ^7^Li signals from globally disparate cores, this strongly suggests that these marine carbonates are recording original seawater δ^7^Li values, with a carbonate fractionation factor.

### Data treatment and availability

We updated the depth-age models for Sites 1263 and U1333 to the GTS2020^27^; this was also done for Site 702 from ref. ^18^, such that the proxy data (see Supplementary Information) we plot our model results against are also updated. The tiepoints used for this can be found in Supplementary Table 1. The isotopic data for all three sites can be found in Supplementary Tables 2–4, which can be found in the accompanying Excel file and is also available for download from the Figshare data repository at 10.5522/04/23749197. Supplementary Tables 2 and 3 also include trace element data for Sites 1263 and U1333. The isotopic data for Sites 1263 and U1333 are plotted in Figs. 1 and 2, and the Site 959 data are plotted in Supplementary Fig. 1.

To make it easier to compare the general trend of the isotopic data with our modelling results we combined the data points from Sites 1263 and U1333, and using MATLAB’s curve fitting app we applied a smoothing spline to the average data points and also to the fully propagated uncertainties of ±2*σ* to create a window of possible isotopic values for our timeframe of interest (41.5–39.0 Ma). For both the lithium and osmium isotope data, the piecewise polynomial is computed from *p*, which was set to 0.999, and *x* was normalized by the mean of 40.17 with a *σ-*value of 0.5538. The individual isotopic data for Sites 1263 and U1333 and the results from the smoothing treatment applied to the combined sites (as seen in Fig. 2) can also be found in a bundle with the model code (see the Model section below).

### Model

The biogeochemical box model we use in this study (CARLIOS) is an adaptation of the CARMER model^71^, which was used to investigate carbon and mercury cycling during the Permo–Triassic mass extinction event, but has also been used to investigate the influence of calcium (via evaporites) on climate in the Cenozoic^72^; thus, no processes specific to the Permo–Triassic mass extinction are included. Here, we strip out the mercury cycle and incorporate the lithium and osmium cycles (Supplementary Fig. 4). The operation of the lithium and osmium cycles is not based on the mercury cycle and, in this model, neither of these elemental cycles have a direct influence on the carbon cycle. Another introduction to this model is the inclusion of a simple silicon cycle. This enables us to investigate the influence of reverse weathering (that is, the formation of marine authigenic clays) on the carbon and lithium cycles.

The model has a crust and three ocean boxes (consisting of a low-latitude surface ocean (s), a high-latitude surface ocean (h) and a deep ocean (d), to enable the thermohaline mixing of oceanic components (for example, dissolved inorganic carbon)), as well as an atmosphere box. The carbon, silicon and osmium cycles also have atmosphere boxes, which enable air–sea gas exchange for CO_2_ and the deposition of dust (for example, silicon-bearing aeolian, and osmium-bearing aeolian and cosmic) to the two surface ocean boxes. For simplicity, the schematic for the osmium cycle combines the dust fluxes and hydrothermal fluxes (Supplementary Fig. 4c), but these are treated separately within the model itself.

An important distinction from the CARMER model is that we do not assume that carbonates precipitate (that is, the burial flux of carbonates) entirely out of low-latitude shallow waters. Instead, since pelagic calcifiers had evolved by this point, much of the carbonate burial takes place in the deep ocean with some proportion of total carbonate burial taking place in warm shelf environments, following ref. ^73^. In addition, we do not assume that the calcium carbonate solubility product has the same value for the different ocean boxes and that it was the same during the MECO as it is during the present day. Instead this is calculated throughout, following the equation utilized in ref. ^74^ and using the possible oceanic concentration of magnesium at ~40 Ma (refs. ^40,75^). For the lithium cycle the following are adopted: the parameterization of ref. ^76^ is followed to derive the flux size and the δ^7^Li signature of rivers at time *t*; the present-day flux sizes and isotope ratios are used from refs. ^37,67^; and the split of the sinks between marine authigenic clays and the alteration of oceanic crust is used from ref. ^20^. In all scenarios we include in our modelling a temperature-dependent effect on the lithium isotope fractionation factor during uptake into secondary minerals (both marine and terrestrial), which, as with other warming events, will decrease the δ^7^Li of rivers (δ^7^Li_riv_)^36,77^. To compare our model results with our δ^7^Li_carb_ data, we applied a Δ^7^Li_sw-carb_ of 3–5‰ (the dark grey band in Fig. 3m) and 2–6‰ (the light grey band in Fig. 3m) to the average δ^7^Li_sw_ produced by the model, to convert it to a δ^7^Li_carb_ record^28–31^.

We run the model forwards in time and start the model runs at 55 Ma. We run the model from 55 Ma so that the reservoirs can reach a steady state (in terms of the magnitude and isotopic values) before introducing perturbations to the system at the various time points indicated in the different scenarios detailed in the main text and the Supplementary Information. We highlight the fact that this is not a steady-state model (that is, we are not setting the derivatives in Supplementary Table 15 to zero). CARLIOS is run 5,000 times as a Monte Carlo simulation, with the model choosing values within defined ranges for certain parameters (see Supplementary Table 13). Table 1 lists the parameter changes for each scenario.

CARLIOS was constructed in MATLAB using a desktop PC with an eight-core Intel Core i7-9700 CPU @ 3.00 GHz. The model uses MATLAB’s parallel computing toolbox to utilize all eight cores to decrease the run time (typically <2 h for 5,000 model runs).

### Further information on the successful model Scenario 8

We include here some additional rationale behind the decision to model a decrease in [Mg]_sw_ and an increase in unradiogenic osmium sourced from continental eruptions for Scenario 8. There is evidence of increasing glauconite (as seen at Site 959), smectite and palygorskite clays during the approximate MECO timeframe^46,55,78^, and other research suggests that, generally, the magnitude of reverse weathering (a notable sink of magnesium) increases as temperatures increase^38,79,80^. This may be a reason why [Mg]_sw_ has increased by ~15 mmol over the past ~40 Myr, as the deep ocean cooled and the reverse weathering uptake of magnesium reduced, although a global reorganisation of the silicon cycle may have also played a part^55,81^. Combined, this information implies that the formation of marine clays during the MECO increased, taking up more magnesium and reducing its concentration in seawater. The LOWESS (locally weighted scatterplot smoothing) bootstrapping of data does hint at a decline in the Mg/Ca ratio during the MECO, as data from a ridge-flank CaCO_3_ vein trends towards a low ratio, with coral data showing a large post-MECO increase^82^. The Mg/Ca data from the study used for our CO_2_ proxy data (see Supplementary Information for CO_2_ data)^18^ indicate minor decreases in Mg/Ca across the ‘warming phase’ of the MECO, with an increase at the MECO peak, although the absolute values of Mg/Ca vary substantially across the four ODP sites sampled. It may be that clay formation (marine and terrestrial) altered the Mg/Ca ratio during the warming phase (resulting in the steady accumulation of CO_2_ in the atmosphere and resultant warming), but a pulse of additional CO_2_ input to the atmosphere during the peak MECO led to a large amount of congruent weathering, releasing more magnesium to the oceans. Eventually this would have aided in carbonate formation, but on a shorter timescale resulted in the sharp decline in pH^18^. Post-MECO, as the deep ocean began to cool, the reverse weathering flux would have started to decline, leading to less CO_2_ formation.

Although silicon is also trapped by clays, thermal expansion of the oceans due to the warming of the climate may have increased sand-grain dissolution^83^. Therefore, although clays would have retained some silicon liberated during weathering, this may have been balanced, at least for a while, by enhanced quartz dissolution. It has been shown that aragonite dissolution helps to preserve calcite deposited below the CCD as it is more soluble, yet Mg-calcites are even more soluble^84^. Thus, if there is a reduction in magnesium in the oceans, there is likely to be a reduction in magnesium calcites precipitating and then being transported to the seafloor. This may then reduce the protection from dissolution given to aragonite—and subsequently calcite—below the CCD, and hence the decline in the CaCO_3_ content of marine sedimentary rocks deposited during the MECO^12^. This may have continued until the surface layers were covered in clays, reducing the CaCO_3_ exposure to acidity^74^. These changes to ocean chemistry will undoubtedly have had effects on the pH and nutrient availability, and thus may have had some influence on ocean biota, but not large enough to cause extinction crises. Indeed, although there is evidence of change, there is no record of large-scale turnover, and benthic foraminifera records suggest that environmental conditions after the MECO returned to their pre-MECO state^11,85–88^.

For the osmium cycle, simple mass-balance calculations indicate that the ^187^Os/^188^Os of rivers must have been considerably less radiogenic than they are today (at 1.2–1.5)^34^ to achieve the ^187^Os/^188^Os_initial_ signature of the pre-MECO seawater^16^. Remnants of previous large igneous provinces (LIPs) were in or transited through the humid zone before the MECO^89^ (Supplementary Fig. 5) and the ^187^Os/^188^Os_initial_ of seawater post-emplacement of these LIPs shows distinctly unradiogenic values (for example, ref. ^90^). It is possible that osmium was leached from rocks during weathering of the LIPs and was then subsequently trapped by clay-associated organic matter. This osmium was probably then released during clay dissolution in the humid zone, and thus we surmise that the weathering of these LIPs supplied unradiogenic osmium (compared with the present day), even when accounting for increasing radiogenicity due to the time elapsed since emplacement. This is supported by the relatively stable ^87^Sr/^86^Sr values before the MECO as well^91^. For the MECO itself, Scenarios 1–6 suggest that enhanced weathering, either due to greater $p_{{\mathrm{CO}}_{2}}$ levels or erosion, is not sufficient to replicate the excursion seen in the data, and thus in Scenarios 7 and 8 we invoked a moderate increase (~75% of the present-day combined hydrothermal fluxes) in the delivery of osmium to the ocean with a mantle-like ^187^Os/^188^Os, as data from various LIPs suggest that eruptive material was very unradiogenic^90^. However, the trend towards a more unradiogenic seawater signal could instead have been the result of a diminished silicate weathering flux^16^ or a mixture of the two processes. As we do not prescribe any short-lived changes within the MECO timeframe, our model misses the small mid-MECO increase in ^187^Os/^188^Os_initial_, which may have occurred due to greater weathering of more radiogenic materials or a temporary decrease in the amount of mafic-associated volcanism.

## Online content

Any methods, additional references, Nature Portfolio reporting summaries, source data, extended data, supplementary information, acknowledgements, peer review information; details of author contributions and competing interests; and statements of data and code availability are available at 10.1038/s41561-023-01234-y.

## Supplementary information

Supplementary Information Supplementary Fig. 1–5, Discussion, Tables 7–15 and refs. 1–78.

Supplementary Tables Supplementary Tables 1–6.

## Data Availability

All the data supporting the results of this study can be found in the Supplementary Information associated with this manuscript and are also available from the Figshare data repository at 10.5522/04/23749197.

## Code availability

The model schematic plus further information on some of the parameters, the full model equations and the parameter values required to recreate the model can be found in the Supplementary Information, and the MATLAB version of the model is freely available from the Zenodo repository at 10.5281/zenodo.8027661.

## References

[1] Scotese CR, Song H, Mills BJW, van der Meer DG (2021). Phanerozoic paleotemperatures: the Earth’s changing climate during the last 540 million years. Earth Sci. Rev..

[2] Gaskell DE (2022). The latitudinal temperature gradient and its climate dependence as inferred from foraminiferal δ^18^O over the past 95 million years. Proc. Natl Acad. Sci. USA.

[3] Westerhold T (2020). An astronomically dated record of Earth’s climate and its predictability over the last 66 million years. Science.

[4] Zachos JC, Dickens GR, Zeebe RE (2008). An early Cenozoic perspective on greenhouse warming and carbon-cycle dynamics. Nature.

[5] Zachos J, Pagani H, Sloan L, Thomas E, Billups K (2001). Trends, rhythms, and aberrations in global climate 65 Ma to present. Science.

[6] Lourens LJ (2005). Astronomical pacing of late Palaeocene to early Eocene global warming events. Nature.

[7] Walker JCG, Hays PB, Kasting JF (1981). A negative feedback mechanism for the long-term stabilization of Earth’s surface temperature. J. Geophys. Res..

[8] Zeebe RE (2012). History of seawater carbonate chemistry, atmospheric CO_2_, and ocean acidification. Annu. Rev. Earth Planet. Sci..

[9] Bohaty SM, Zachos JC (2003). Significant Southern Ocean warming event in the late middle Eocene. Geology.

[10] Bohaty, S. M., Zachos, J. C., Florindo, F. & Delaney, M. L. Coupled greenhouse warming and deep-sea acidification in the middle Eocene. *Paleoceanogr.*10.1029/2008PA001676 (2009).

[11] Bijl PK (2010). Transient middle Eocene atmospheric CO_2_ and temperature variations. Science.

[12] Sluijs A, Zeebe RE, Bijl PK, Bohaty SM (2013). A middle Eocene carbon cycle conundrum. Nat. Geosci..

[13] van der Ploeg R (2023). North Atlantic surface ocean warming and salinization in response to middle Eocene greenhouse warming. Sci. Adv..

[14] Cramwinckel MJ (2018). Synchronous tropical and polar temperature evolution in the Eocene. Nature.

[15] Rivero-Cuesta L (2019). Paleoenvironmental changes at ODP Site 702 (South Atlantic): anatomy of the Middle Eocene Climatic Optimum. Paleoceanogr. Paleoclimatol..

[16] van der Ploeg R (2018). Middle Eocene greenhouse warming facilitated by diminished weathering feedback. Nat. Commun..

[17] Zachos JC (2005). Rapid acidification of the ocean during the Paleocene–Eocene Thermal Maximum. Science.

[18] Henehan, M. J. et al. Revisiting the Middle Eocene Climatic Optimum ‘carbon cycle conundrum’ with new estimates of atmospheric *p*CO_2_ from boron isotopes. *Paleoceanogr. Paleoclimatol.*10.1029/2019PA003713 (2020).

[19] Brantley SL, Shaughnessy A, Lebedeva MI, Balashov VN (2023). How temperature-dependent silicate weathering acts as Earth’s geological thermostat. Science.

[20] Misra S, Froelich PN (2012). Lithium isotope history of Cenozoic seawater: changes in silicate weathering and reverse weathering. Science.

[21] Dellinger M (2015). Riverine Li isotope fractionation in the Amazon River basin controlled by the weathering regimes. Geochim. Cosmochim. Acta.

[22] Pogge von Strandmann PAE, Frings PJ, Murphy MJ (2017). Lithium isotope behaviour during weathering in the Ganges Alluvial Plain. Geochim. Cosmochim. Acta.

[23] Bouchez J, Von Blanckenburg F, Schuessler JA (2013). Modeling novel stable isotope ratios in the weathering zone. Am. J. Sci..

[24] Sparks, D. L. *Environmental Soil Chemistry* 2nd edn, 187–205 (Academic, 2003); 10.1016/B978-012656446-4/50006-2

[25] Kennedy MJ, Löhr SC, Fraser SA, Baruch ET (2014). Direct evidence for organic carbon preservation as clay–organic nanocomposites in a Devonian black shale; from deposition to diagenesis. Earth Planet. Sci. Lett..

[26] Schlesinger, W. H. & Bernhardt, E. S. *Biogeochemistry: An Analysis of Global Change* 4th edn, 99–139 (Academic, 2020); 10.1016/B978-0-12-814608-8.00004-9

[27] Gradstein, F. M. et al. (eds) *Geologic Time Scale 2020* (Elsevier, 2020).

[28] Marriott CS, Henderson GM, Crompton R, Staubwasser M, Shaw S (2004). Effect of mineralogy, salinity, and temperature on Li/Ca and Li isotope composition of calcium carbonate. Chem. Geol..

[29] Pogge von Strandmann PAE, Jenkyns HC, Woodfine RG (2013). Lithium isotope evidence for enhanced weathering during Oceanic Anoxic Event 2. Nat. Geosci..

[30] Pogge von Strandmann, P. A. E., Dellinger, M. & West, A. J. *Lithium Isotopes: A Tracer of Past and Present Silicate Weathering* (Cambridge Univ. Press, 2021); 10.1017/9781108990752

[31] Pogge von Strandmann PAE (2019). Assessing bulk carbonates as archives for seawater Li isotope ratios. Chem. Geol..

[32] Füger A (2022). Effect of growth rate and pH on Li isotope fractionation during its incorporation in calcite. Geochim. Cosmochim. Acta.

[33] Charrieau LM (2023). Controls on lithium incorporation and isotopic fractionation in large benthic foraminifera. Minerals.

[34] Lu X, Kendall B, Stein HJ, Hannah JL (2017). Temporal record of osmium concentrations and ^187^Os/^188^Os in organic-rich mudrocks: iImplications for the osmium geochemical cycle and the use of osmium as a paleoceanographic tracer. Geochim. Cosmochim. Acta.

[35] Pogge von Strandmann PAE (2017). Global climate stabilisation by chemical weathering during the Hirnantian glaciation. Geochem. Perspect. Lett..

[36] Pogge von Strandmann PAE (2021). Lithium isotope evidence for enhanced weathering and erosion during the Paleocene–Eocene Thermal Maximum. Sci. Adv..

[37] Pogge von Strandmann PAE, Kasemann SA, Wimpenny JB (2020). Lithium and lithium isotopes in Earth’s surface cycles. Elements.

[38] Isson TT, Planavsky NJ (2018). Reverse weathering as a long-term stabilizer of marine pH and planetary climate. Nature.

[39] Gislason SR, Arnorsson S, Armannsson H (1996). Chemical weathering of basalt in wouthwest Iceland: effects of runoff, age of rocks and vegetative/glacial cover. Am. J. Sci..

[40] Lowenstein, T. K., Kendall, B. & Anbar, A. D. in *Treatise on Geochemistry* 2nd edn, Vol. 8 (eds Holland, H. D. & Turekian, K. K.) 569–622 (Elsevier, 2013).

[41] Hay WW (2006). Evaporites and the salinity of the ocean during the Phanerozoic: implications for climate, ocean circulation and life. Palaeogeogr. Palaeoclimatol. Palaeoecol..

[42] Miller KG (2020). Cenozoic sea-level and cryospheric evolution from deep-sea geochemical and continental margin records. Sci. Adv..

[43] Methner K (2016). Rapid middle Eocene temperature change in western North America. Earth Planet. Sci. Lett..

[44] Fernández DA, Palazzesi L, González Estebenet MS, Tellería MC, Barreda VD (2021). Impact of mid Eocene greenhouse warming on America’s southernmost floras. Commun. Biol..

[45] Baatsen M (2020). The middle to late Eocene greenhouse climate modelled using the CESM 1.0.5. Clim. Past.

[46] Rego ES (2018). Mineralogical evidence for warm and dry climatic conditions in the Neo-Tethys (eastern Turkey) during the middle Eocene. Palaeogeogr. Palaeoclimatol. Palaeoecol..

[47] Torres MA, West AJ, Li G (2014). Sulphide oxidation and carbonate dissolution as a source of CO_2_ over geological timescales. Nature.

[48] Cramwinckel MJ (2020). Surface-circulation change in the southwest Pacific Ocean across the Middle Eocene Climatic Optimum: inferences from dinoflagellate cysts and biomarker paleothermometry. Clim. Past.

[49] Wanner C, Sonnenthal EL, Liu XM (2014). Seawater δ^7^Li: a direct proxy for global CO_2_ consumption by continental silicate weathering?. Chem. Geol..

[50] Siewert J, Ferlito C (2008). Mechanical erosion by flowing lava. Contemp. Phys..

[51] Pruski FF, Nearing MA (2002). Climate-induced changes in erosion during the 21st century for eight U.S. locations. Water Resour. Res..

[52] Nearing MA (2005). Modeling response of soil erosion and runoff to changes in precipitation and cover. Catena.

[53] Pogge von Strandmann PAE, Henderson GM (2015). The Li isotope response to mountain uplift. Geology.

[54] Dunlea AG (2017). Cenozoic global cooling and increased seawater Mg/Ca via reduced reverse weathering. Nat. Commun..

[55] Cornaggia F (2020). Abyssal oceanic circulation and acidification during the Middle Eocene Climatic Optimum (MECO). Sci. Rep..

[56] Gaillardet J, Dupré B, Louvat P, Allègre CJ (1999). Global silicate weathering and CO_2_ consumption rates deduced from the chemistry of large rivers. Chem. Geol..

[57] Giorgioni M (2019). Carbon cycle instability and orbital forcing during the Middle Eocene Climatic Optimum. Sci. Rep..

[58] Spofforth, D. J. A. et al. Organic carbon burial following the Middle Eocene Climatic Optimum in the central western Tethys. *Paleoceanogr.*10.1029/2009PA001738 (2010).

[59] Lyle, A. O. & Lyle, M. W. Missing organic carbon in Eocene marine sediments: is metabolism the biological feedback that maintains end-member climates? *Paleoceanogr.*10.1029/2005PA001230 (2006).

[60] Regaudie-De-Gioux, A. & Duarte, C. M. Temperature dependence of planktonic metabolism in the ocean. *Global Biogeochem. Cycles*10.1029/2010GB003907 (2012).

[61] Scotese, C. R. *PALEOMAP PaleoAtlas for GPlates and the PaleoData Plotter Program* (PALEOMAP Project, 2016); http://www.earthbyte.org/paleomap-paleoatlas-for-gplates/

[62] van der Meer DG (2017). Reconstructing first-order changes in sea level during the Phanerozoic and Neoproterozoic using strontium isotopes. Gondwana Res..

[63] Rae JWB (2021). Atmospheric CO_2_ over the past 66 million years from marine archives. Annu. Rev. Earth Planet. Sci..

[64] Scotese CR (2021). An atlas of Phanerozoic paleogeographic maps: the seas come in and the seas go out. Annu. Rev. Earth Planet. Sci..

[65] Westerhold T, Röhl U (2013). Orbital pacing of Eocene climate during the Middle Eocene Climate Optimum and the chron C19r event: missing link found in the tropical western Atlantic. Geochem., Geophys. Geosyst..

[66] Dellinger M (2020). The effects of diagenesis on lithium isotope ratios of shallow marine carbonates. Am. J. Sci..

[67] Kalderon-Asael B (2021). A lithium-isotope perspective on the evolution of carbon and silicon cycles. Nature.

[68] Pogge von Strandmann PAE (2011). Variations of Li and Mg isotope ratios in bulk chondrites and mantle xenoliths. Geochim. Cosmochim. Acta.

[69] Pogge von Strandmann PAE (2019). Experimental determination of Li isotope behaviour during basalt weathering. Chem. Geol..

[70] Lechler M, Pogge von Strandmann PAE, Jenkyns HC, Prosser G, Parente M (2015). Lithium-isotope evidence for enhanced silicate weathering during OAE 1a (Early Aptian Selli event). Earth Planet. Sci. Lett..

[71] Dal Corso J (2020). Permo–Triassic boundary carbon and mercury cycling linked to terrestrial ecosystem collapse. Nat. Commun..

[72] Shields GA, Mills BJW (2021). Evaporite weathering and deposition as a long-term climate forcing mechanism. Geology.

[73] Walker JCG, Kasting JF (1992). Effects of fuel and forest conservation on future levels of atmospheric carbon dioxide. Palaeogeogr. Palaeoclimatol. Palaeoecol..

[74] Zeebe RE (2012). LOSCAR: Long-term Ocean–atmosphere–Sediment CArbon cycle Reservoir model v2.0.4. Geosci. Model Dev..

[75] Horita J, Zimmermann H, Holland HD (2002). Chemical evolution of seawater during the Phanerozoic. Geochim. Cosmochim. Acta.

[76] Caves Rugenstein JK, Ibarra DE, von Blanckenburg F (2019). Neogene cooling driven by land surface reactivity rather than increased weathering fluxes. Nature.

[77] Li G, West AJ (2014). Evolution of Cenozoic seawater lithium isotopes: coupling of global denudation regime and shifting seawater sinks. Earth Planet. Sci. Lett..

[78] Baldermann A (2022). Impact of green clay authigenesis on element sequestration in marine settings. Nat. Commun..

[79] Couture RA (1977). Composition and origin of palygorskite-rich and montmorillonite-rich zeolite-containing sediments from the Pacific Ocean. Chem. Geol..

[80] Thiry M, Pletsch T (2011). Palygorskite clays in marine sediments: records of extreme climate. Dev. Clay Sci..

[81] Higgins JA, Schrag DP (2015). The Mg isotopic composition of Cenozoic seawater – evidence for a link between Mg-clays, seawater Mg/Ca, and climate. Earth Planet. Sci. Lett..

[82] Evans D (2018). Eocene greenhouse climate revealed by coupled clumped isotope-Mg/Ca thermometry. Proc. Natl Acad. Sci. USA.

[83] Fabre, S., Jeandel, C., Zambardi, T., Roustan, M. & Almar, R. An overlooked silica source of the modern oceans: are sandy beaches the key? *Front. Earth Sci.*10.3389/feart.2019.00231 (2019).

[84] Sulpis O (2022). Aragonite dissolution protects calcite at the seafloor. Nat. Commun..

[85] Moebius I, Friedrich O, Edgar KM, Sexton PF (2015). Episodes of intensified biological productivity in the subtropical Atlantic Ocean during the termination of the Middle Eocene Climatic Optimum (MECO). Paleoceanogr..

[86] D’Onofrio R (2021). Impact of the Middle Eocene Climatic Optimum (MECO) on foraminiferal and calcareous nannofossil assemblages in the Neo-Tethyan Baskil section (eastern Turkey): paleoenvironmental and paleoclimatic reconstructions. Appl. Sci..

[87] Boscolo Galazzo F, Giusberti L, Luciani V, Thomas E (2013). Paleoenvironmental changes during the Middle Eocene Climatic Optimum (MECO) and its aftermath: the benthic foraminiferal record from the Alano section (NE Italy). Palaeogeogr. Palaeoclimatol. Palaeoecol..

[88] Boscolo Galazzo F, Thomas E, Giusberti L (2015). Benthic foraminiferal response to the Middle Eocene Climatic Optimum (MECO) in the south-eastern Atlantic (ODP Site 1263). Palaeogeogr. Palaeoclimatol. Palaeoecol..

[89] Johansson L, Zahirovic S, Müller RD (2018). The interplay between the eruption and weathering of large igneous provinces and the deep-time carbon cycle. Geophys. Res. Lett..

[90] Dickson, A. J., Cohen, A. S. & Davies, M. in *Large Igneous Provinces: A Driver of Global Environmental and Biotic Changes* (eds Ernst, R. E. et al.) 229–246 (Wiley, 2021); 10.1002/9781119507444.ch10

[91] McArthur, J. M., Howarth, R. J., Shields, G. A. & Zhou, Y. in *Geologic Time Scale 2020* (eds Gradstein, F. M. et al.) 211–238 (Elsevier, 2020); 10.1016/b978-0-12-824360-2.00007-3

