# Publisher Correction: Enhanced clay formation key in sustaining the Middle Eocene Climatic Optimum

**DOI:** 10.1038/s41561-023-01280-6

**Published:** 2023-09-07

**Authors:** Alexander J. Krause, Appy Sluijs, Robin van der Ploeg, Timothy M. Lenton, Philip A. E. Pogge von Strandmann

**Affiliations:** 1 University College London, Earth Sciences, London, UK; 2 Department of Earth Sciences, Faculty of Geosciences, Utrecht University, Utrecht, The Netherlands; 3 Shell Global Solutions International B.V., Amsterdam, The Netherlands; 4 Global Systems Institute, University of Exeter, Exeter, UK; 5 Institute of Geosciences, Johannes Gutenberg University, Mainz, Germany

**Keywords:** Carbon cycle, Palaeoclimate, Element cycles, Geochemistry, Palaeoceanography

Correction to: *Nature Geoscience* 10.1038/s41561-023-01234-y, published online 31 July 2023.

In the version of the article originally published, a reference was missing from the seventh paragraph of the “A global shift towards enhanced clay formation” section and the first paragraph of the “Further information on the successful model Scenario 8” section (in the latter instance, the reference is cited in the added text “although a global reorganisation of the silicon cycle may have also played a part”). The reference—Dunlea, A. G. et al. Cenozoic global cooling and increased seawater Mg/Ca via reduced reverse weathering. *Nat. Commun*. **8**, 844 (2017)—has now been inserted as new ref. 54. In the “Data treatment and availability section”, the isotopic data, which can be found in the Figshare data repository at 10.5522/04/23749197, were incorrectly said to be found in the PANGAEA data repository. These corrections have been made in the HTML and PDF versions of the article.

