# Supplementary material for: Enhanced clay formation key in sustaining the Middle Eocene Climatic Optimum
Source: Nat Geosci. 2023 Jul 31;16(8):730–8. doi: 10.1038/s41561-023-01234-y (PMC10409649; doi:10.1038/s41561-023-01234-y)
Supplement: Supplementary file 1 — Supplementary Fig. 1–5, Discussion, Tables 7–15 and refs. 1–78. [file 41561_2023_1234_MOESM1_ESM.pdf]

nature geoscience

Article

https://doi.org/10.1038/s41561-023-01234-y

Enhanced clay formation key in sustaining the Middle Eocene Climatic Optimum

In the format provided by the authors and unedited

Site 959

The sediments of Site 959 for the interval explored in this study are principally composed of various forms of porcellanite, with fluctuations in carbonate content as the dominant lithology cycles through the following types: porcellanite foram calcareous chalk, clayey porcellanite, porcellanite micrite chalk, micritic porcellanite, porcellanite with micrite, and porcellanite. For depths associated with the MECO there is also evidence that the sediments are relatively organic-rich, with some dissemination of glauconite and pyrite[1]. The $^{187}Os/^{188}Os_{initial}$ values at Site 959 are marginally more radiogenic than the other two sites and the isotope excursion appears to begin earlier, but these differences are not substantial enough to suggest that Os dynamics operated differently at Site 959 compared to Sites 1263 and U1333. Site 959 also exhibits a positive LIE (Fig. S1), moving from a low of ~8‰ prior to the MECO, to a high of ~15‰ during the MECO. The excursion begins before the start of the MECO as defined by TEX_{86} data[2] and has a longer duration. However, there has been an assumption that changes to the TEX_{86} data should be co-eval with changes to the $\delta^{18}O$ record, but this may not necessarily be true and non-thermal factors may have influenced the record[3,4]. As such Site 959 could have a MECO duration similar to the other two sites, but the age constraints for this site are very uncertain, thus we do not assign specific ages to the data from this site. More importantly, our δ^7Li data for this site show a much lower starting value, pre-MECO, to the other two sites, with an excursion that is approximately double in size (see compared to Fig. 1). As the sediments from all three sites were deposited in open, deep ocean settings at ~40 Ma, a possible reason for the difference in δ^7Li data at Site 959 may be that the porcelaneous nature of the sediments has induced a lithium isotope fractionation factor very different from that typical of bulk carbonates, charting at the extreme end of that seen in biogenic aragonites[5]. Hence, we focused the bulk of our main text discussion on Sites 1263 and U1333.

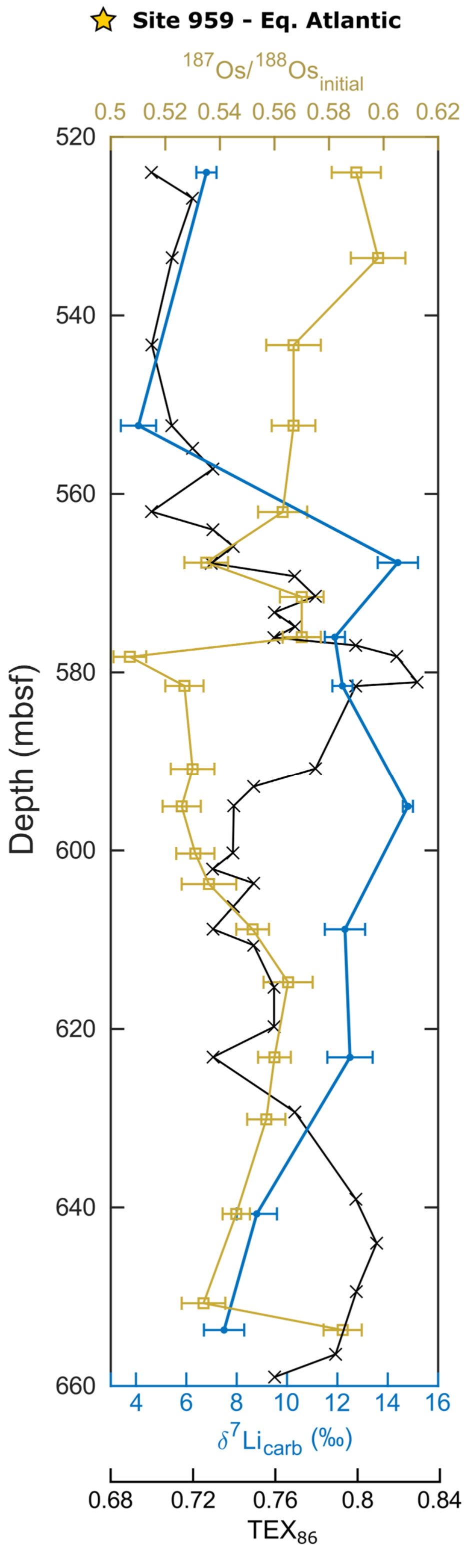

Figure S1 | Isotopic data for ODP Site 959. *δ^7Li_{carb} values are in blue (this study) with the filled circles representing the mean of each sample (n = 3) and the error bars represent the ± 2SD precision, $^{187}Os/^{188}Os_{initial}$ values are in gold*[6].

Temperature and CO_2 proxies

For ground-truthing our model outputs, it is crucial to compare them to proxy data. Alongside the δ^7Li_{carb} and $^{187}Os/^{188}Os_{initial}$ data from this study and van der Ploeg *et al.*[6], we also compare our model results against pCO_2 and temperature proxy data. Atmospheric CO_2 proxy data for the MECO comes almost exclusively from one study[7], with only one data point from stomatal indices and one data point from alkenones, from just before and just after the MECO, respectively, available[8]. The pCO_2 data points (see Fig. S2) are based on $\delta^{11}B$ from planktic foraminifera from ODP Sites 865, 1260, 1263 and 702. The sites were aligned and ages reported on the 2012 GTS[7,9] but we have updated the ages to the 2020 GTS[10]. The authors converted $\delta^{11}B$ to a pH and then, using their best estimate of $\delta^{11}B_{sw}$ = 38.5 – 38.9‰, converted pH to pCO_2 values, assuming either a constant ocean saturation state ($\Omega_{calcite}$) or a constant ocean alkalinity (ALK), which represents, approximately, the two pCO_2 endmembers[7]. Although there are uncertainty ranges for the data (see their Fig. 7)[7], we do not include these in our figures, as our aim is to approximately match the timing and general magnitude inferred from the $\delta^{11}B$ data.

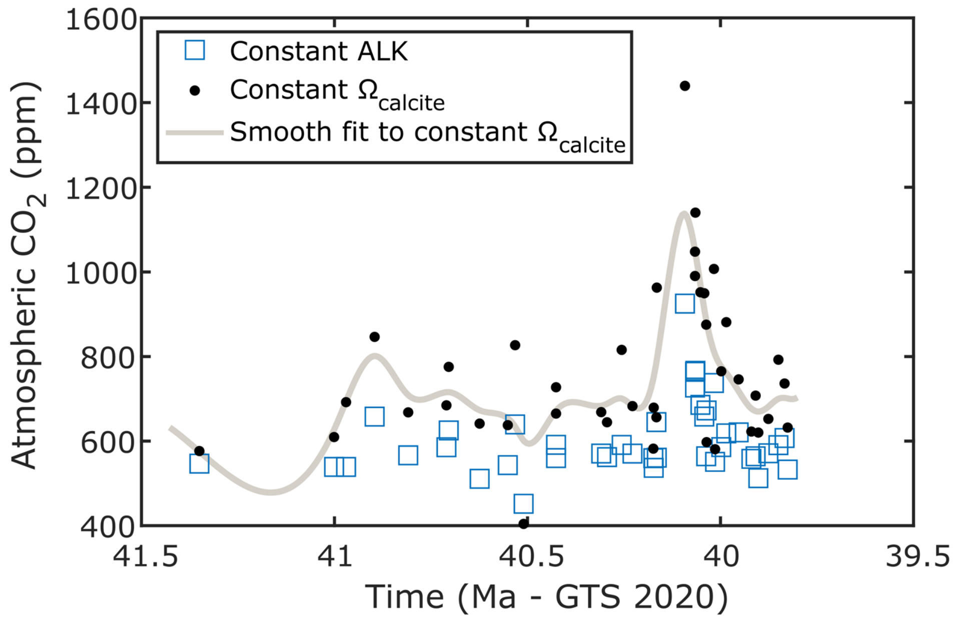

Figure S2 | Atmospheric CO_2 concentrations during the MECO. *The concentrations are from Henehan et al.*[7]*, where they calculated CO_2 by using their best estimate of $\delta^{11}B_{sw}$ obtained from foraminifera and assumed either a constant calcite saturation (black dots) or a constant alkalinity (blue squares). The grey line is the result of using MATLAB's curve fitting app to fit a smoothing spline to the CO_2 estimations from constant calcite saturation and is plotted here to show the general trend of the changes to CO_2.*

For the temperature proxy data, we use the $\delta^{18}O$ data from benthic foraminifera (ODP Sites 865 and 1260) and from bulk sediments (ODP Sites 1263 and 702), which Henehan *et al.*[7] collated and tied to the 2012 GTS[9], and as with the $\delta^{11}B$ data, we have updated to the 2020 GTS[10] (see Fig. S3). Hansen *et al.*[11] derived a set of equations that can be used to convert $\delta^{18}O$ data to temperature, with different equations to be employed when the Earth was ice-free (and therefore $\delta^{18}O$ values are <1.75‰) and when the Earth had ice sheets and thus changes in $\delta^{18}O$ reflect changes to the ice mass as well as temperature ($\delta^{18}O$ data is >1.75‰). As all the $\delta^{18}O$ data here[7] is <1.75‰, we convert $\delta^{18}O$ to surface temperatures following Hansen *et al.*[11] using a surface temperature of 22.07°C at 40.884 Ma:

$$Temp_{deep\ ocean} = -4 * \delta^{18}O + 12 \quad (1)$$

$$Temp_{surface}(t) = Temp_{surface}(t-1) + \left[Temp_{deep\ ocean}(t) - Temp_{deep\ ocean}(t-1)\right] \quad (2)$$

We then used MATLAB's curve fitting app to fit a smoothing spline to our data (see Fig. 3b). Although Henehan *et al.*[7] generated $\delta^{18}O$ data from their planktic foraminifera samples, we do not use these in this study because even in the modern day it is difficult to disentangle seasonal changes to shell $\delta^{18}O$ from depth migration effects[12], thus making it more difficult to convert $\delta^{18}O$ to surface temperatures.

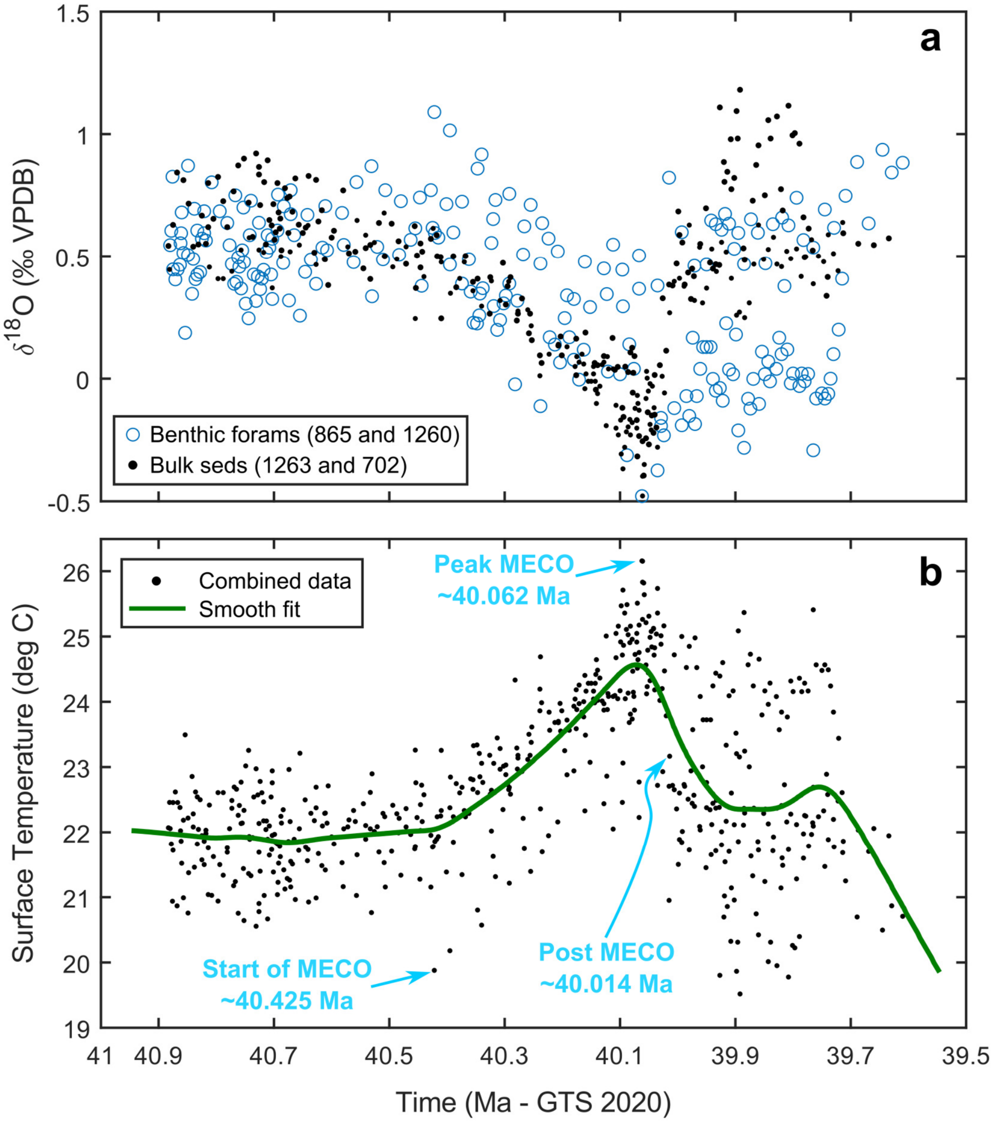

Figure S3 | Global deep ocean δ^{18}O data across the MECO and the resultant estimated surface temperature. *In a) δ^{18}O data is from benthic foraminifera at ODP Sites 865 and 1260, and from bulk sediments at ODP Sites 1263 and 702, with ages tied to the 2020 Geologic Time Scale[7,10]. See text for more details. In b) δ^{18}O data is converted to a global surface temperature using the equations from Hansen et al.[11], and a smoothing spline fit to the data (green line) is added. The 'start', 'peak' and 'post' MECO times are based on the δ^{18}O data from Site 702[7].*

The CARLIOS model

Schematic

The schematic for the CARLIOS model can be seen in Fig. S4. Much of the information about the model can be found in the Methods section of the main text, however we also discuss some of the key parameters below.

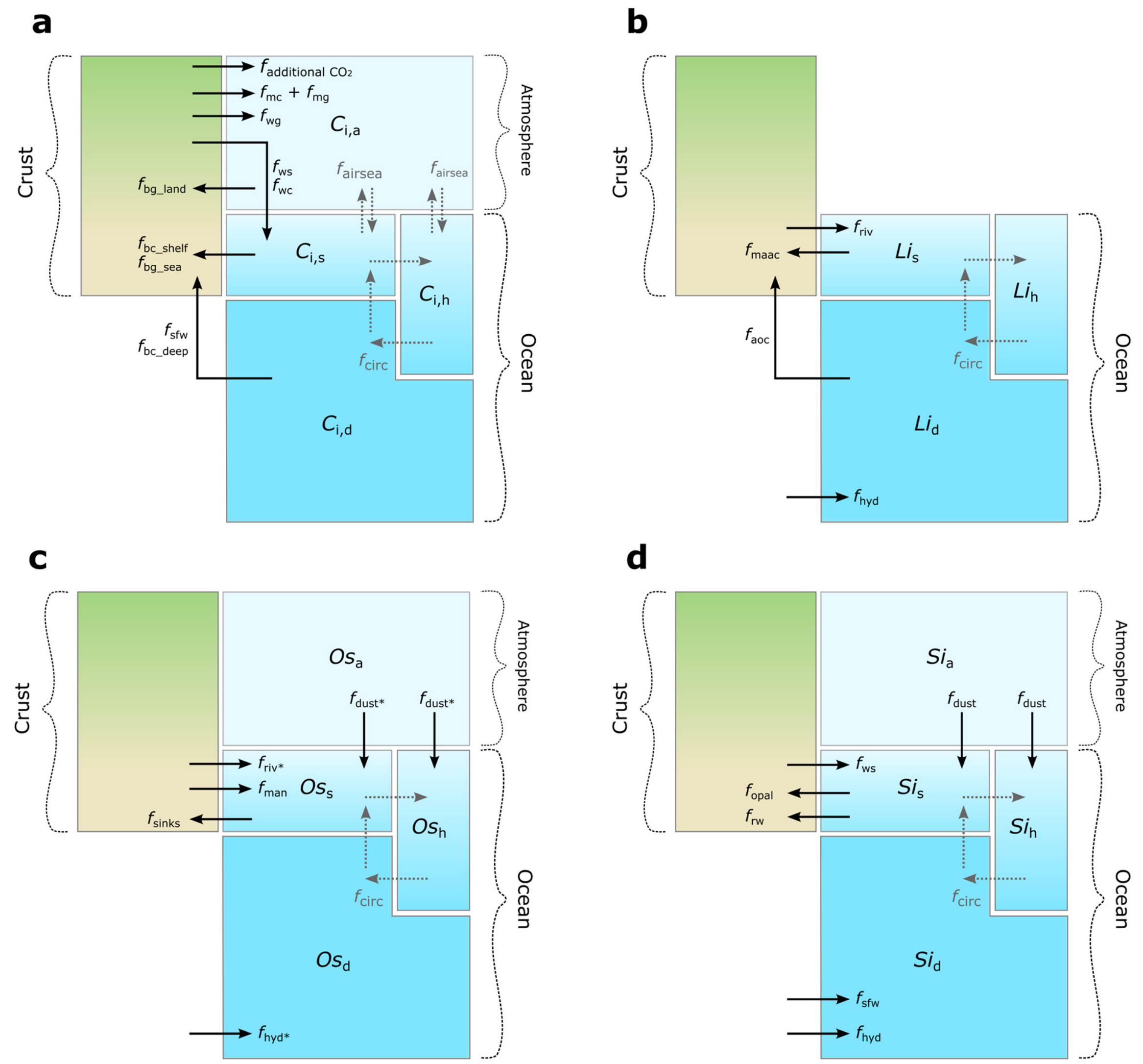

Figure S4 | Schematic of the CARLIOS model. *Panel a) The carbon cycle, b) the lithium cycle, c) the osmium cycle. The ocean is split into three boxes: a low latitude surface ocean (s), a high latitude surface ocean (h) and a deep ocean box (d), and a thermohaline circulation (f_{circ}) mixes components between the boxes. We assume lithium has no atmospheric component in this model. In a) an air-sea gas exchange mixes carbon between the atmosphere and surface ocean boxes, degassing supplies inorganic (f_{mc}) and organic carbon (f_{mg}) to the atmosphere, as does the oxidation of organic carbon (f_{wg}), while the weathering of silicates (f_{ws}) and carbonates (f_{wc}) transfers carbon from the atmosphere and land to the ocean. The burial of carbonates on the shelf (f_{bc_shelf}) and in the deep ocean (f_{bc_deep}), seafloor weathering (f_{sfw}) and terrestrial and marine organic carbon (f_{bg_land}, f_{bg_sea}) returns carbon to the sediments. In b) lithium is supplied to the oceans by rivers (f_{riv}) and hydrothermal fluids (f_{hyd}) and is returned to the sediments via marine authigenic clay formation (f_{maac}) and alteration of oceanic basalt (f_{aoc}). In c) the sources of Os include the dust flux (f_{dust}), which includes both cosmic and aeolian dust, the riverine flux (f_{riv}) which includes that from rivers and groundwaters, the hydrothermal flux (f_{hyd}) including both low-temperature and high-temperature hydrothermal activity, and the terrestrial weathering of juvenile crust (f_{man}). The sinks of Os are combined (f_{sinks}). In d) silicate weathering (f_{ws}), aeolian dust (f_{dust}), seafloor weathering (f_{sfw}) and hydrothermal fluids (f_{hyd}) deliver silicon to the ocean and the two sinks are via reverse weathering (f_{rw}) and SiO_2 formation (amorphous and opal, f_{opal}).*

Key model parameters

Long-term tectonic degassing

The long-term tectonic degassing rate (fG) is a significant variable in long-term box models[13–15], controlling the return rate of carbon (and sulfur etc.) species from the crust to the ocean/atmosphere via both degassing and metamorphism. This parameter, based on seafloor spreading rates, has also been used in simple box modelling to control the rate of hydrothermal input/output of other elements, such as lithium or strontium[14,16]. In terms of CO_2 input to the exogenic system (i.e. oceans and atmosphere), basing fG on seafloor spreading rates likely does not fully encapsulate the effects of continental rifting

and continental arc volcanism, both of which can be significant sources of CO_2 to the atmosphere, especially if lithospheric or crustal carbonates are remobilised as a result[17–19]. Indeed, incorporation of continental rift lengths into fG has been shown to somewhat close the gap between $p$$CO_2$ estimates from geochemical proxies and those generated by models such as GEOCARBSULF[20,21] for the Cenozoic. Such detailed deep carbon cycling work is beyond the scope of this study. Instead, fG prior to the MECO is estimated to be 1.25 – 1.43x the present-day level based on seafloor spreading rates[13,22] but tectonic degassing may have been up to 3x the present-day level when rift lengths are taken into consideration[23]. The use of the upper value of the range (when taking into account the additional atmospheric CO_2 (see below)) allows our model to produce a reasonable fit to pre-MECO CO_2 and temperature estimates, and the input of elements into the ocean sourced from seafloor spreading also results in a general match to our isotopic proxy data. However, because of the uncertainty range on seafloor spreading rates, and tectonic degassing in general, we incorporate fG into our Monte Carlo simulations with bounds of 1.25 – 1.65x the present-day level. We then model different timings and amounts of additional CO_2 input directly to the atmosphere.

Additional atmospheric CO_2 injection estimates

Prior to and during the MECO, volcanic CO_2 degassing from the India – Asia collision (in Tibet – the Himalayas), may have been over an order of magnitude higher than is presently released in this area[18]. It has also been hypothesised that a flare up of continental arc volcanism in the Neotethys suture zone (in what is present day Iran and Azerbaijan) could have been a trigger for the MECO[19]. Elsewhere along the Neotethys suture zone, the volcanic arc extends westwards (into what is present day Armenia, Georgia and Turkey)[24], and there is evidence for regional uplift and a switch from submarine to subaerial volcanism (which is important for increasing CO_2 degassing to the atmosphere without supplying isotopically light δ^7Li to the ocean) from the Lutetian to Bartonian Ages[25]. There is also a minor increase in the areal extent of continental large igneous province (LIP) eruptions from 41 to 40 Ma[26], which may be linked to some initial eruption of flood basalts in Southern Ethiopia/Northern Kenya prior to the main eruptive phase of the Afar-Arabian LIP[27,28]. While some of the CO_2 degassed

from these locations may have been accounted for in fG, we assume that overall fG is an underestimate of the total amount of CO_2 delivered to the atmosphere from tectonic activity and thus model an additional input of CO_2. For each scenario (ScX, where X indicates the scenario number) we assume that there is a baseline of additional CO_2 which is steadily degassing from Tibet[18], and we incorporate the uncertainty regarding the magnitude of this flux into our Monte Carlo model simulations. Then for Sc1 and Sc6-8 we model a further injection of CO_2 into the atmosphere during the MECO sourced, primarily, from Iran[19], but also possibly from other volcanic arcs and continental LIPs as described above. Unlike for Iran, however, we currently have no estimations as to how much CO_2 may have degassed from other volcanic arcs during this time.

Long-term uplift

The long-term uplift of sediments and their subsequent erosion (fR) is another key variable in biogeochemical box models. Based on sediment deposition reconstructions, with a smoothing of 100 Myrs, during the mid-Eocene the long-term uplift value is estimated to be approximately 0.5x the present-day[29,30]. Increasing uplift is a common feature in inter-eruptive or pre-eruptive stages for volcanoes[31], while eruptions themselves increase erosion, either via lavas or lahars[32]. Additionally, due to increasing atmospheric CO_2 levels (and thus temperature), rainfall patterns, magnitudes and intensity can all change to produce large variations in erosion on geologically short timescales that may not be evidenced in the long-term record[33,34]. Thus, we might expect an increase in erosion due to an increase in volcanic activity, alongside complex topographical changes in Tibet producing some uplift[35] and for example, evidence for intermittent but substantial uplift in the Isle of Wight[36]. Also, at 40 Ma some of the remaining LIP materials were in areas of higher altitude (Fig. S5), such as northern South America, the Alps, China and Mongolia, and parts of Tibet, meaning that some volume of basaltic rock would have been uplifted to zones more susceptible to high rates of erosion and/or weathering. As such, we model increases in fR in Sc2 and Sc6-8.

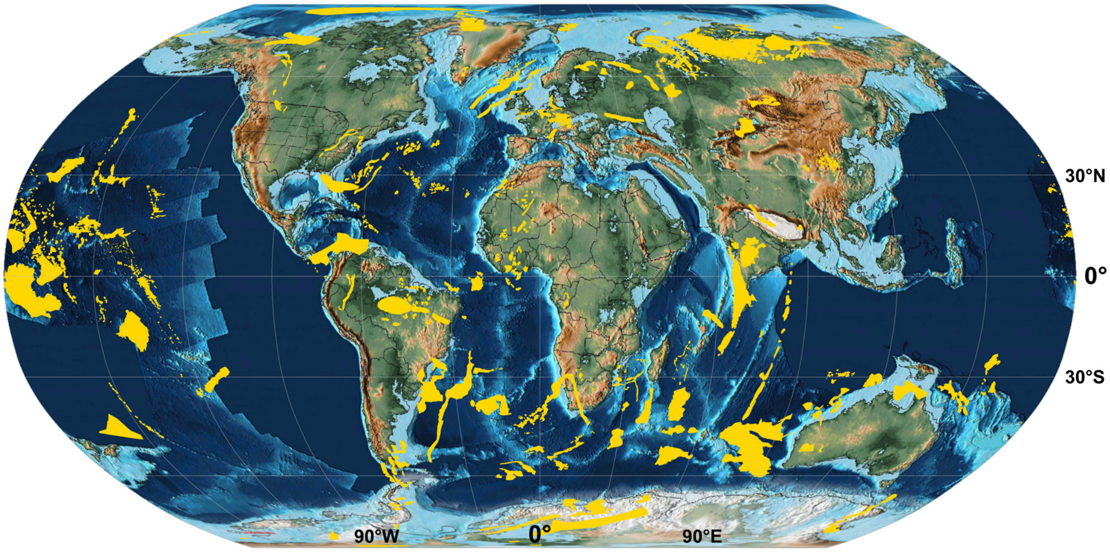

Figure S5 | Vestigial large igneous provinces during the MECO. *The paleogeographic distribution of LIP-associated sediments (in yellow) remaining from previous emplacements, at ~40 Ma. This map was made in GPlates with the base paleogeography from Scotese*[37] *and the LIP distribution from ref.* [26] *and refs. therein.*

In Sc8 (our preferred scenario, see main text) and Sc7 (see below) we set the minimum value for the pre-MECO to 0.5x and the maximum value during the MECO to 1x present day uplift. These values are chosen as the sediment reconstruction and smoothing of the data[29,30], as well as a separate polynomial fit to terrigenous sediments[38] indicate that the long-term fR has not exceeded ~1.1x the present-day rate throughout the Phanerozoic, albeit rapid erosion events are likely missed out in the data. Although it may appear as if we are utilising a small increase in erosion, if the pre-anthropogenic erosion flux is ~$2x10^{10}$ tonnes yr^{-1} [39–41], then we are modelling an increase of $1x10^{10}$ tonnes yr^{-1} at the peak of the MECO. We do, however, model two scenarios (Sc2 and Sc6) whereby fR reaches a peak of 3x present-day to test what a large erosion event might do to the Earth system.

The erosion to silicate weathering ratio, δ^7Li_{riv} and FLi_{riv}

In their modelling, Caves-Rugenstein *et al.*[42] calculate the lithium isotopic signature of rivers (δ^7Li_{riv}) and the riverine flux of Li (FLi_{riv}) dynamically. They estimate a weathering intensity (W/D, where W is weathering and D is denudation, which is the total of erosion (E) + W) by first converting the model silicate weathering flux from moles yr^{-1} into tonnes yr^{-1}. Using: this flux; the normalised uplift (fR); and a global erosion to weathering ratio (E:W), they produce an erosion flux in tonnes yr^{-1}. They then use their W/D values to generate a value for δ^7Li_{riv}. The δ^7Li_{riv} can then be used to derive a value for the fraction of lithium partitioned from the bedrock into the dissolved load via a Rayleigh distillation function, which is then used to calculate FLi_{riv}. An uncertainty in this multistep approach is the present day global silicate weathering flux[43] which is used to convert between Mol yr^{-1} to tonnes yr^{-1}. However, the magnitude of silicate weathering is typically outweighed by the erosion flux when calculating denudation rates, such that at the present day a 28% reduction in the silicate weathering rate only decreases total denudation[41,43,44] from $2.25x10^{10}$ tonnes yr^{-1} to $2.04x10^{10}$ tonnes yr^{-1}. A more significant unknown is the global erosion to weathering ratio (E:W), which is only used in the Li and not the C cycle calculations. It is assumed that E:W at 16 Ma ranges between 3–40[42,45], but this may have been different at ~40 Ma, as fR is lower during the Eocene than the Neogene[29,30].

As our data show a rise in the δ^7Li_{sw} it is likely that there was some increase in the riverine δ^7Li (some of the seawater excursion may have also been sourced from changes to the Li sinks – e.g. see Extended Data Fig. 3), but without modelling we do not know whether the Earth moved from a kinetically-limited, or a supply-limited version of congruent weathering, to incongruent weathering[46–48]. We used the parameterisation of Caves-Rugenstein *et al.*[42] to calculate the δ^7Li_{riv} and FLi_{riv} dynamically, but for Sc1-6 we kept E:W fixed throughout the model runs at a value of 5 which we found generated a pre-MECO δ^7Li_{riv} signature that leads to the model δ^7Li_{carb} record being consistent with our sample data (see Extended Data Figs. 1 – 7m). When combined with large increases in fR (such as in Sc2 and Sc6), this produces a reasonable fit to the δ^7Li_{carb} data. For Sc7 and Sc8 we included the E:W as a variable in the Monte Carlo simulation, setting the boundaries to incorporate the full Neogene range (3–40)[42,45] and used a more modest increase in fR which produces a very good fit to the δ^7Li_{carb} data.

Additional information on the failed scenarios – Extended data figures 1 – 7

In Extended Data Figs. 1 – 7 we plot the key parameter changes plus model results for Sc1-7: the failed scenarios. Some of the scenario hypotheses (e.g. Sc1 and Sc4) were based upon previous work[49] but parameter values for, as an example, the input of additional CO_2 to the atmosphere (Sc1) or a near-total shutdown in marine organic carbon (C_{org}) burial were chosen to try and match our $p$$CO_2$ model outputs to the proxy data. Table 1 in the main text illustrates why these model runs are considered failures (e.g. the lysocline does not shoal, or the model does not reproduce the δ^7Li_{carb} data etc.). In Sc3 we apply a scaling to the reverse weathering flux to artificially force an increase in its magnitude, while holding fR and the additional CO_2 input at their pre-MECO values. This scenario was conducted to test the effect of a pulse of reverse weathering (and thus a large increase of marine authigenic clay (MAAC) formation) on the modelled estimate for δ^7Li_{sw} (and thus δ^7Li_{carb}). However, we do not fix the riverine flux or its δ^7Li signature in this scenario, instead it varies in response to the CO_2 released to the atmosphere from reverse weathering reactions. Likewise, in scenarios such as Sc1 or Sc2 where we change the additional CO_2 input or the fR value, which affects terrestrial weathering and erosion, we do not hold the lithium sink fluxes (MAAC formation and the alteration of oceanic crust) at a constant size. Rather, these fluxes vary in response to the amount of silicon in the ocean. Thus, in Fig. 3o in the main text, and Extended Data Figs. 1 – 7o, the rivers versus sinks panel reflects changes to both the riverine input and the combined outputs (both flux size and isotopic value) in each modelled scenario, but showcases whether the rivers or the combined sinks hold the greater control over the δ^7Li_{sw} record in our model (<1 for sinks, >1 for rivers).

Calculating changes to Mg and Ca retention in soils

Our modelling, based on the W/D results (Fig. 4), suggests the Earth transitioned (pre-MECO) from a global regime with a mix of secondary mineral formation and dissolution, with dissolution slightly outweighing formation, to one more dominated by secondary mineral formation (even if only slightly) during the MECO. It is possible that the early Cenozoic warmth[50] coupled with the eruption of large igneous provinces (e.g. the North Atlantic Igneous Province)[51], which generated lots of highly weatherable basalt, resulted in high rates of weathering and erosion but eventually in widespread secondary mineral (e.g. clay) formation well before the MECO. Over time, as clays became thicker and much of the basalt from the LIPs was eroded, the Earth slowly transitioned, globally, to a more supply limited regime, where clays started to dissolve. As noted in the main text, there will have been spatial heterogeneity, with some areas experiencing high erosion and low chemical weathering (and vice versa). This ties in with the δ^7Li record of seawater in the Paleogene[52] where δ^7Li_{sw} rises by a few ‰ after the Early Eocene Climatic Optimum (~50 Ma) to ~44 Ma whereafter the limited available data suggests δ^7Li_{sw} starts to decline and is backed up by our low δ^7Li pre-MECO data.

The present day global δ^7Li_{riv} average is 23‰[48] and the W/D, based on estimates of total rock weathering[44] of 2.13×10^9 t yr^{-1} and total erosion[41] of 2×10^{10} t yr^{-1}, is 0.096 (see main text Fig. 4a), although the weathering estimate does not include contributions from oceanic island basalts. It has been proposed that the silicate weathering flux may have been overestimated[43], however, at most, this reduces W/D to 0.090. Meanwhile, a modelled estimate for W/D for the pre-anthropogenic present is ~0.063, but the δ^7Li_{riv} is higher at ~28‰[42]. Our modelling suggests that during the MECO, the global δ^7Li_{riv} average was ~23–24‰ with a W/D of ~0.065–0.085, which is very similar to the present day. We attempt to work out what the change in weathering regime may have meant for the amount of Ca and Mg cations retained on land, and whether such an increase is feasible.

The general trend of soil formation rates appear to follow changes in climate over time, with higher rates occurring during periods of higher temperatures[53]. Assuming that surface temperatures have declined by 6°C over the last 50 Myrs[54–56], while the soil formation rate[53] has declined from 2.2×10^{10} to

1.3×10^{10} tonnes yr^{-1}, we calculate an increase in soil formation at the MECO peak to $2.280x10^{10}$ tonnes yr^{-1} compared to the pre-MECO background formation rate (~1.830×10^{10} tonnes yr^{-1}), assuming a global average surface temperature increase of 3°C[57].

If the Earth's land surface is approximately 148,940,000 km^2 and assuming that approximately 94% of this area could host soil formation[58], with a soil depth of 2 m, this gives an approximate value of 280,007,200 km^3 or $2.800x10^{17}$ m^3 soil volume, split as $4.200x10^{16}$ m^3 topsoil and $2.380x10^{17}$ m^3 subsoil. A tonne of a standard, moist soil, equals approximately 0.670 m^3 thus at the present day, there is $6.269x10^{16}$ tonnes of topsoil and $3.552x10^{17}$ tonnes of subsoil ($4.179x10^{17}$ tonnes in total).

To work out how much of the soil is composed of clay-size minerals we use the clay fraction (% of the soil that is composed of clays) per unit area data (2° grid resolution) for the present day Earth[59]. Any values that were less than 0 (indicative of grid cells with no data, such as the ocean) were converted to NaNs in MATLAB. Out of 16,200 cells for the topsoil, 5,117 of these were NaNs (the same was true for the subsoil dataset). The clay fraction data for the topsoil for all grid cells was summed together (see Table S7) and then divided by 11,083 (the number of cells with data, i.e. non-NaNs) to give a global average clay fraction for the topsoil. The same was done with the subsoil dataset.

Table S7 | The clay fraction of soils on the present-day Earth.

Soil	Total clay fraction (% soil)	Average clay fraction (% soil)
**Top**	104438.73	9.42
**Sub**	120401.05	10.86

As the average clay fraction of topsoil and subsoil is 9.42% and 10.86% respectively, using our estimates of soil tonnage, we can thus surmise that clay-size minerals make up $5.905x10^{15}$ tonnes and $3.858x10^{16}$ tonnes respectively ($4.448x10^{16}$ tonnes in total).

However, not all clay-size minerals contain Ca and/or Mg, so we work out the fraction of total clay that is kaolinite, smectite, vermiculite or illite, which are the most abundant clay-size minerals in soils and are Ca or Mg containing[59]. Again, any areas of the Earth with no data are converted to NaNs and omitted. For each mineral type the topsoil and subsoil are summed together, to give a global value (see Table S8). The kaolinite, smectite, vermiculite and illite data are also added together. Using representative formulae for the various minerals, a weight percentage of Mg or Ca can be calculated, and subsequently so can the fraction of Mg or Ca in each clay mineral type.

Table S8 | Fraction of Ca and or Mg in the major clay-size minerals.

	Totals (kg m^{-2})	Fraction of all clays	Formula used	Wt% of Ca or Mg	Fraction of clay type is Ca or Mg
**All clays**	1.383e6	-	-	-	-
**Kaolinite**	3.514e5	25.410 %	$Al_2Si_2O_5(OH)_4$	0.1206 % Mg^	0.030 %
**Smectite***	2.009e5	14.529 %	$Na_{0.2}Ca_{0.1}Al_2Si_4O_{10}(OH)_2(H_2O)_{10}$	0.73 % Ca	0.106 %
**Vermiculite**	1.215e5	8.787 %	$Mg_{1.8}Fe^{2+}_{0.9}Al_{4.3}SiO_{10}(OH)_2{\bullet}4(H_2O)$	8.68 % Mg	0.763 %
**Illite**	3.495e5	25.268 %	$K_{0.6}(H_3O)_{0.4}Al_{1.3}Mg_{0.3}Fe^{2+}_{0.1}Si_{3.5}O_{10}(OH)_2{\bullet}(H_2O)$	1.87 % Mg	0.473 %
**Kaolinite + Smectite + Vermiculite + Illite**	1.023e6	73.994 %	-	-	1.372 %

* As montmorillonite, ^From Tardy *et al.*[60]

Thus, using our tonnage of clays in soils (4.448×10^{16} tonnes) and the percentage of Ca and Mg in clays, we can calculate the amount of these cations in total in soils at Present: 6.103×10^{14} tonnes.

If there was an increase from 0.5x to 1x present day erosion across the MECO (corresponding to 1×10^{10} tonnes yr^{-1} to 2×10^{10} tonnes yr^{-1}), as per our favoured Sc8 (see main text), then using our soil formation rates across the MECO (an increase from ~1.830×10^{10} tonnes yr^{-1} to 2.280×10^{10} tonnes yr^{-1}), this would indicate a change of net soil formation from 0.17×10^{10} tonnes yr^{-1} to 0.28×10^{10} tonnes yr^{-1}. Assuming

that the percentages of the various clay-size minerals, and thus the amount of Ca and Mg, in soils has not changed considerably over time (and we acknowledge that this is an area of considerable uncertainty), we calculate the net addition of Ca and Mg to soils across the MECO would increase from 2.332×10^{7} tonnes yr^{-1} to 3.841×10^{7} tonnes yr^{-1}.

If the MECO lasts 400 kyrs, as per our modelling, using linear interpolation between the two net addition of Ca and Mg values, and assuming they are added via an equally distributed 'witch's hat' shape, the total addition of carbonate forming cations to the soils is 1.235×10^{13} tonnes. If the pre-MECO soil Ca and Mg reservoir was similar to today, then this addition would make the Ca and Mg reservoir equal to 6.227×10^{14} tonnes at the end of the MECO, which represents a ~2% increase in the amount of Ca and Mg retained in soils. Of course, the pre-MECO Ca and Mg soil reservoir may have been much smaller than at Present, which means a greater percentage increase. Alternatively, because the W/D calculated by the model at the MECO peak is approximately equal to that at the present-day (see Fig. 4a), we could deduce that prior to the MECO the terrestrial reservoir of Ca and Mg was equal to 5.98×10^{14} tonnes ($6.103 \times 10^{14} - 1.235 \times 10^{14}$ tonnes). For comparison, the amount of Mg and Ca in seawater is approximately as follows:

Mg: 1.29×10^{21} l * 1.272 g/l = 1.64×10^{21} g, which is 1.64×10^{15} t

Ca: 1.29×10^{21} l * 0.411 g/l = 5.3×10^{20} g, which is 5.3×10^{14} t

Summed together, the total amount is 2.17×10^{15} t which suggests that our estimates for Ca and Mg retained in soils is feasible in the context of the present-day Earth system and also highlights the potential power that clay retention of cations may have on ocean chemistry and climate.

Other possible MECO triggers

Although in this study we have invoked an increase in tectonic activity and associated volcanism to explain the MECO, there are other potential triggers for the MECO that have been suggested. One of these is the influence of orbital changes, as it has been found that the MECO occurs coincidentally with

the minima of ~400 kyr and 2.4 Myr eccentricity cycles[61] and the timing of these correlate to variations in the $\delta^{13}C$ and $\delta^{18}O$ records[62]. It is uncertain as to whether eccentricity changes alone could have triggered the MECO (perhaps via changing ocean and hydrological circulation patterns, affecting weathering and primary productivity rates), or whether they perhaps enhanced the effects of some other trigger, such as enhanced volcanism.

Another possibility is the impact of bolide(s) upon the Earth's surface. As an extraterrestrial body, a bolide would likely contain unradiogenic Os[63] and depending on the lithology of the impact location, could expose silicate minerals to the surface and/or vaporise carbonate rocks. In Eurasia, several bolides potentially of MECO age, have been documented[64], with the Beenchime-Salaaty impact crater exposing Cambrian age carbonate rocks, the Logancha uncovering Triassic basalts, and the Sunak exposing primarily Devonian rhyolites. However, the age constraints on these impact events remains poor. Additionally, bolides have low δ^7Li values[65] which could potentially lower δ^7Li_{sw} dependent on whether the impact site was sufficiently far enough away from the coastline to allow for clay formation and isotopic fractionation. Thus, in light of all available data we favour changes in tectonic activity to explain the MECO.

Full model parameters

Table S9 | Model constants.

Parameter	Algebraic Representation	Value	Source
Thermohaline speed	f_circ_sv	15 Sv	Dal Corso *et al.*[66] and refs. therein
Relative area of low latitude surface ocean	surf_area	0.85	Dal Corso *et al.*[66] and refs. therein
Relative area of high latitude surface ocean	high_area	0.15	Dal Corso *et al.*[66] and refs. therein
Gas exchange timescale	tau	10 yr	Dal Corso *et al.*[66] and refs. therein
Long-term climate sensitivity	gamma	5°C	Mills *et al.*[30]
Switch from shallow to deep ocean carbonate deposition	fC	1	Berner[67]
Basaltic land area	fA_{bas}	1.218	Lenton *et al.*[14]

Granitic land area	fA_{gran}	0.9974	Lenton *et al.*[14]
Present global average surface temperature	$temp_0$	288°K	Berner[67]
Effect of solar insolation on temperature	Ws	7.4°	Berner[68]
Conversion from Sv to m^3 yr^{-1} for thermohaline circulation	th_{conv}	$3.15x10^{13}$	Dal Corso *et al.*[66] and refs. therein
Temperature controlled runoff factor	RUN	0.045	Berner and Kothavala[38]
Proportion of plants fertilised by increasing CO_2	FERT	0.4	Berner and Kothavala[38]
Activation energy for basalt weathering	ACT_{bas}	0.0608	Lenton *et al.*[14]
Activation energy for granite weathering	ACT_{gran}	0.0724	Lenton *et al.*[14]
Activation energy for carbonate weathering	ACT_{carb}	0.087	Lenton *et al.*[14]
Fraction of silicates which are basaltic	basfrac	0.3	Lenton *et al.*[14]
Second dissociation constant	kacid	$7.4x10^{-10}$	Dal Corso *et al.*[66] and refs. therein
Alpha value for calculating the calcite solubility constant	solalpha	0.08333	Zeebe[69]
Present day calcium concentration in the oceans	Ca_conc_0	10 mmol m^{-3}	Lowenstein *et al.*[70]
Present day magnesium concentration in the oceans	Mg_conc_0	53 mmol m^{-3}	Lowenstein *et al.*[70]
Present day [Mg]/[Ca]	Mg_Ca_0	5.3 molar	Lowenstein *et al.*[70]
Magnesium concentration in the oceans during the MECO	Mg_conc_MECO	38.4	Horita *et al.*[71] and Lowenstein *et al.*[70]
Present day carbonate saturation	$Saturation_0$	3	Dal Corso *et al.*[66] and refs. therein
$\delta^{13}C$ fractionation	$alpha_C$	27‰	Dal Corso *et al.*[66] and refs. therein
$\delta^{13}C$ of carbonates	$d13c_c$	0‰	Dal Corso *et al.*[66] and refs. therein
$\delta^{13}C$ of organic carbon	$d13c_g$	-27‰	Dal Corso *et al.*[66] and refs. therein
$\delta^{13}C$ of additional CO_2	$d13c_{input}$	0‰	This study
Present δ^7Li fractionation during clay formation	alpha_Li_maac	20‰	Kalderon-Asael *et al.*[72] and refs. therein
Present δ^7Li fractionation during alteration of oceanic crust	alpha_Li_aoc	13‰	Kalderon-Asael *et al.*[72] and refs. therein
Curve fitting constant	a_li	4.5	Caves-Rugenstein *et al.*[42]
Curve fitting constant	b_li	0.0575	Caves-Rugenstein *et al.*[42]
Average δ^7Li of bulk rock	delta_rock_li	1.5‰	Caves-Rugenstein *et al.*[42]

Fractionation between water and secondary minerals	delta_sec_li	-17‰	Caves-Rugenstein *et al.*[42]
δ^7Li of hydrothermal fluids	delta_hyd_li	8‰	Pogge von Strandmann *et al.*[48]
Fraction of the total Li sink which comes from alteration of oceanic crust	Li_split	0.3	Misra and Froelich[52]
Fraction of riverine Os which comes from silicate weathering	Os_split	0.3	Georg *et al.*[73]
$^{187}Os/^{188}Os$ of high-temperature hydrothermal fluids	delta_hth_os	0.13	Lu *et al.*[63]
$^{187}Os/^{188}Os$ of low-temperature hydrothermal fluids	delta_lth_os	0.88	Lu *et al.*[63]
$^{187}Os/^{188}Os$ of aeolian dust	delta_adust_os	1.1	Lu *et al.*[63]
$^{187}Os/^{188}Os$ of cosmic dust	delta_cdust_os	0.12	Lu *et al.*[63]
$^{187}Os/^{188}Os$ of freshly erupted materials from the mantle	delta_man_os	0.13	Lu *et al.*[63]

*N.B. $^{187}Os/^{188}Os$ of aeolian dust is the average of the range in Lu *et al.*[63].

Table S10 | Present day fluxes.

Parameter	Algebraic Representation	Value	Source
Carbonate degassing	Fmc_0	$12x10^{12}$ Mol yr^{-1}	Dal Corso *et al.*[66]
Carbonate weathering	F_wc_0	$8x10^{12}$ Mol yr^{-1}	Lenton *et al.*[14]
Seafloor weathering	F_sfw_0	$1.75x10^{12}$ Mol yr^{-1}	Mills *et al.*[74]
Carbonate burial	F_bc_0	$18.25x10^{12}$ Mol yr^{-1}	Dal Corso *et al.*[66]
Silicate weathering	F_ws_0	$10.25x10^{12}$ Mol yr^{-1}	Dal Corso *et al.*[66]
Granite weathering	F_wgran_0	$7.175x10^{12}$ Mol yr^{-1}	Dal Corso *et al.*[66]
Basalt weathering	F_wbas_0	$3.075x10^{12}$ Mol yr^{-1}	Dal Corso *et al.*[66]
Organic carbon degassing	Fmg_0	$1.25x10^{12}$ Mol yr^{-1}	Lenton *et al.*[14]
Organic carbon weathering	F_wg_0	$7.75x10^{12}$ Mol yr^{-1}	Lenton *et al.*[14]
Marine organic carbon burial	F_bg_sea_0	$4.5x10^{12}$ Mol yr^{-1}	Lenton *et al.*[14]
Terrestrial organic carbon burial	F_bg_land_0	$4.5x10^{12}$ Mol yr^{-1}	Lenton *et al.*[14]
Hydrothermal flux of lithium	F_riv_Li_0	$8x10^{9}$ Mol yr^{-1}	Pogge von Strandmann *et al.*[48]
Riverine flux of lithium	F_hyd_Li_0	$6x10^{9}$ Mol yr^{-1}	Pogge von Strandmann *et al.*[48]

Combined sinks of lithium	F_sink_Li_0	14×10^{9} Mol yr^{-1}	Pogge von Strandmann *et al.*[48]
Combined riverine and groundwater flux of osmium	F_riv_Os_0	2405 Mol yr^{-1}	Lu *et al.*[63]
High temperature hydrothermal flux of osmium	F_hth_Os_0	157.7 Mol yr^{-1}	Lu *et al.*[63]
Low temperature hydrothermal flux of osmium	F_lth_Os_0	197.14 Mol yr^{-1}	Lu *et al.*[63]
Cosmic dust flux of osmium	F_cdust_Os_0	176.1 Mol yr^{-1}	Lu *et al.*[63]
Aeolian dust flux of osmium	F_adust_Os_0	147.2 Mol yr^{-1}	Lu *et al.*[63]
Subaerial weathering of freshly erupted mantle flux of osmium	F_mantle_Os_0	1.0813 Mol yr^{-1}	Lu *et al.*[63]
Combined sinks of osmium	F_sinks_Os_0	3084.22 Mol yr^{-1}	This study (all sources combined)
Hydrothermal input of calcium	F_hyd_Ca_0	1.8×10^{12} Mol yr^{-1}	Farkas *et al.*[75]
Hydrothermal input of silicon	F_hyd_Si_0	0.6×10^{12} Mol yr^{-1}	Tréguer and De La Rocha[76]
Aeolian dust input of silicon	F_adust_Si_0	0.5×10^{12} Mol yr^{-1}	Tréguer and De La Rocha[76]
Uptake of silicon during reverse weathering	F_rw_Si_0	1.75×10^{12} Mol yr^{-1}	Slightly more than Tréguer and De La Rocha[76] but less than Rahman *et al.*[77] – equal to seafloor weathering
Biogenic sink of silicon	F_opal_0	9.6×10^{12} Mol yr^{-1}	This study (all sources combined minus reverse weathering)

*N.B. Osmium fluxes are taken to be the average of the range from Lu *et al.*[63].

Table S11 | Initial reservoir sizes.

Parameter	Algebraic Representation	Value	Source
Low latitude surface ocean volume	$Water_s$	3.07×10^{16} m^3	Dal Corso *et al.*[66] and refs. therein
High latitude surface ocean volume	$Water_h$	1.35×10^{16} m^3	Dal Corso *et al.*[66] and refs. therein

Deep ocean volume	$Water_d$	$1.35x10^{18}$ m^3	Dal Corso *et al.*[66] and refs. therein
Total ocean volume	$Water_t$	$1.3942x10^{18}$ m^3	Dal Corso *et al.*[66] and refs. therein
Present day atmospheric CO_2	A_0	$5x10^{16}$ Mol	Dal Corso *et al.*[66] and refs. therein
Low latitude surface ocean DIC	DIC_s_0	$6x10^{16}$ Mol	Dal Corso *et al.*[66] and refs. therein
High latitude surface ocean DIC	DIC_h_0	$3x10^{16}$ Mol	Dal Corso *et al.*[66] and refs. therein
Deep ocean DIC	DIC_d_0	$3x10^{18}$ Mol	Dal Corso *et al.*[66] and refs. therein
Low latitude surface ocean alkalinity	ALK_s_0	$6x10^{16}$ Mol	Dal Corso *et al.*[66] and refs. therein
High latitude surface ocean alkalinity	ALK _h_0	$3x10^{16}$ Mol	Dal Corso *et al.*[66] and refs. therein
Deep ocean alkalinity	ALK _d_0	$3x10^{18}$ Mol	Dal Corso *et al.*[66] and refs. therein
Low latitude surface ocean calcium	Ca_s_0	$3.1x10^{17}$ Mol	This study (based on global ocean concentration of 10mM)
High latitude surface ocean calcium	Ca_h_0	$1.4x10^{17}$ Mol	As above
Deep ocean calcium	Ca_d_0	$1.4x10^{19}$ Mol	As above
Low latitude surface ocean lithium	Li_s_0	$8.02x10^{14}$ Mol	This study (based on a global ocean concentration of 0.2ppm[78] converted to Mol)
High latitude surface ocean lithium	Li_h_0	$3.52x10^{14}$ Mol	As above
Deep ocean lithium	Li_d_0	$3.52x10^{16}$ Mol	As above
Low latitude surface ocean osmium	Os_s_0	$1.59x10^{6}$ Mol	This study (based on a global ocean concentration of 10 pg/kg[63] converted to Mol)
High latitude surface ocean osmium	Os_h_0	$6.97x10^{5}$ Mol	As above
Deep ocean osmium	Os_d_0	$6.97x10^{7}$ Mol	As above
Low latitude surface ocean silicon	Si_s_0	$2.14x10^{15}$ Mol	This study (based on Tréguer and De La Rocha[76])
High latitude surface ocean silicon	Si_h_0	$9.39x10^{14}$ Mol	As above
Deep ocean silicon	Si_d_0	$9.39x10^{16}$ Mol	As above

Table S12 | Initial isotopic values.

Parameter	Algebraic Representation	Value	Source
$\delta^{13}C$ of the atmosphere	d13c_a_0	-6.5‰	Dal Corso *et al.*[66] and refs. therein
$\delta^{13}C$ of low latitude surface ocean DIC	d13c_DICs_0	2‰	Dal Corso *et al.*[66] and refs. therein
$\delta^{13}C$ of high latitude surface ocean DIC	d13c_DICh_0	2‰	Dal Corso *et al.*[66] and refs. therein
$\delta^{13}C$ of deep ocean DIC	d13c_DICd_0	2‰	Dal Corso *et al.*[66] and refs. therein
$\delta^{7}Li$ of low latitude surface ocean	d7Li_s_0	26‰	This study
$\delta^{7}Li$ of high latitude surface ocean	d7Li_h_0	26‰	This study
$\delta^{7}Li$ of deep ocean	d7Li_d_0	26‰	This study
$^{187}Os/^{188}Os$ of low latitude surface ocean	dOs_s_0	0.515‰	This study / van der Ploeg *et al.*[6]
$^{187}Os/^{188}Os$ of high latitude surface ocean	dOs_h_0	0.515‰	This study / van der Ploeg *et al.*[6]
$^{187}Os/^{188}Os$ of deep ocean	dOs_d_0	0.515‰	This study / van der Ploeg *et al.*[6]

Table S13 | Monte-Carlo simulation parameters.

Parameter	Algebraic Representation	Lower Limit	Upper Limit	Non-Monte-Carlo Value
Global erosion to silicate weathering ratio used to help convert normalised uplift into tonnes yr^{-1}	E:W	3	40	5
Background input of CO_2 directly to the atmosphere from the Tibet region	Tibet	$3.51x10^{12}$ Mol yr^{-1}	$6.61x10^{12}$ Mol yr^{-1}	$5.06x10^{12}$ Mol yr^{-1}
Seafloor spreading rate around the time of the MECO	fG	1.25	1.65	1.45
Present day fraction of Li partitioned from the bedrock into the dissolved load	ray_frac_0	0.2823	0.2994	0.29085
Temperature dependent isotopic fractionation of Li during clay formation	clayfrac	-0.075‰	-0.125‰	-0.1125‰

(marine and terrestrial) and alteration of oceanic crust				
Riverine value of $^{187}Os/^{188}Os$	dosriv	0.49	0.52	0.505
Unradiogenic Os exhumed during a volcanic eruption which is weathered and transported to the ocean	osman	177.42 Mol yr^{-1}	354.84 Mol yr^{-1}	1.0813 Mol yr^{-1}

Full model equations

Table S14 | Flux and parameter equations.

Normalised atmospheric CO_2	$RCO_2 = \frac{CO_2(t)}{CO_2(0)}$	(3)
CO_2 in ppm	$CO_2atm = 280 * RCO_2$	(4)
Split total oceanic concentrations at present day/model initiation into surface, high, and deep components	$X_{j\_0} = X_0 * \frac{water_j}{water_t}$	(5)
Convert reservoirs at present day/model initiation from moles to mol/m^3	$Xconc_{j\_0} = \frac{X_{j\_0}}{water_j}$	(6)
Convert reservoirs (at time = t) from moles to mol/m^3	$Xconc_j = \frac{X_j}{water_j}$	(7)
Thermohaline circulation (m^3 yr^{-1})	$f_{circ} = f_{circ\_sv} * th_{conv}$	(8)
Low latitude surface to high latitude surface ocean DIC transfer	$DIC_{s2h} = f_{circ} * DICconc_s$	(9)
High latitude surface to deep ocean DIC transfer	$DIC_{h2d} = f_{circ} * DICconc_h$	(10)
Deep to low latitude surface ocean DIC transfer	$DIC_{d2s} = f_{circ} * DICconc_d$	(11)
Low latitude surface to high latitude surface ocean ALK transfer	$ALK_{s2h} = f_{circ} * ALKconc_s$	(12)

High latitude surface to deep ocean ALK transfer	$ALK_{h2d} = f_{circ} * ALKconc_h$	(13)
Deep to low latitude surface ocean ALK transfer	$ALK_{d2s} = f_{circ} * ALKconc_d$	(14)
Low latitude surface to high latitude surface ocean Li transfer	$Li_{s2h} = f_{circ} * Liconc_s$	(15)
High latitude surface to deep ocean Li transfer	$Li_{h2d} = f_{circ} * Liconc_h$	(16)
Deep to low latitude surface ocean ALK transfer	$Li_{d2s} = f_{circ} * Liconc_d$	(17)
Low latitude surface to high latitude surface ocean Os transfer	$Os_{s2h} = f_{circ} * Osconc_s$	(18)
High latitude surface to deep ocean Os transfer	$Os_{h2d} = f_{circ} * Osconc_h$	(19)
Deep to low latitude surface ocean ALK transfer	$Os_{d2s} = f_{circ} * Osconc_d$	(20)
Low latitude surface to high latitude surface ocean Ca transfer	$Ca_{s2h} = f_{circ} * Caconc_s$	(21)
High latitude surface to deep ocean Ca transfer	$Ca_{h2d} = f_{circ} * Caconc_h$	(22)
Deep to low latitude surface ocean Ca transfer	$Ca_{d2s} = f_{circ} * Caconc_d$	(23)
Low latitude surface to high latitude surface ocean Si transfer	$Si_{s2h} = f_{circ} * Siconc_s$	(24)
High latitude surface to deep ocean Si transfer	$Si_{h2d} = f_{circ} * Siconc_h$	(25)
Deep to low latitude surface ocean Si transfer	$Si_{d2s} = f_{circ} * Siconc_d$	(26)
Global average surface temperature	$GAST = temp_0 + \left(gamma * \frac{\log RCO_2}{\log 2}\right) - \left(Ws * \frac{time\_myr}{-570}\right)$	(27)

Low latitude surface ocean temperature	$temp_s = 298 + \left(\frac{2}{3} * (GAST - temp_0)\right)$	(28)
High latitude surface ocean temperature	$temp_h = max(275.5 + (GAST - temp_0), 271)$	(29)
Deep ocean temperature	$temp_d = max(275.5 + (GAST - temp_0), 271)$	(30)
Change in GAST from present day	$atemp_{change} = GAST - temp_0$	(31)
Change in low latitude surface ocean temperature from present	$stemp_{change} = temp_s - 298$	(32)
Change in deep ocean temperature from present	$dtemp_{change} = temp_d - 275.5$	
Temperature dependency for basalt weathering	$fT_{bas} = e^{(ACT_{bas} * (GAST - temp_0))} * \left(1 + RUN * (GAST - temp_0)\right)^{0.65}$	(33)
Temperature dependency for granite weathering	$fT_{gran} = e^{(ACT_{gran} * (GAST - temp_0))} * \left(1 + RUN * (GAST - temp_0)\right)^{0.65}$	(34)
Temperature dependency for carbonate weathering	$fT_{carb} = 1 + \left(ACT_{carb} * (GAST - temp_0)\right)$	(35)
Temperature dependency for seafloor weathering	$fT_{sfw} = e^{(ACT_{bas} * (temp_d - 271))}$	(36)
CO_2 dependency for all weathering	$fCO_2 = \left(\frac{2 * RCO_2}{1 + RCO_2}\right)^{FERT}$	(37)
Combined CO_2 and temperature dependency for basalt weathering	$fB_{bas} = fT_{bas} * fCO_2$	(38)
Combined CO_2 and temperature dependency for granite weathering	$fB_{gran} = fT_{gran} * fCO_2$	(39)
Combined CO_2 and temperature dependency for carbonate weathering	$fB_{carb} = fT_{carb} * fCO_2$	(40)
Basalt weathering	$Fwbas = fB_{bas} * Fwbas_0$	(41)
Granite weathering	$Fwgran = fB_{gran} * fR^{0.33}\, Fwgran_0$	(42)
Total silicate weathering	$Fws = Fwbas + Fwgran$	(43)
Seafloor weathering	$Fsfw = fT_{sfw} * fG * Fsfw_0$	(44)

Carbonate weathering	$Fwc = fB_{carb} * fR^{0.9} * Fwc_0$	(45)
Organic carbon weathering	$Fwg = RO_2^{0.5} * fR^{0.33} * Fwg_0$	(46)
Carbonate degassing	$Fmc = fC * fG * Fmc_0$	(47)
Organic carbon degassing	$Fmg = fG * Fmg_0$	(48)
Carbonate equilibrium constants	$kcarb_j = 5.75 \cdot 10^{-4} + \left(6 \cdot 10^{-6} * \left(temp_j - 278\right)\right)$	(49)
CO_2 equilibrium constants	$kCO_{2j} = 0.035 + \left(0.0019 * \left(temp_j - 278\right)\right)$	(50)
Numerator for dissolved bicarbonate	$bicarb_{num} = DICconc_j - \left\{DICconc_j^2 - \left[ALKconc_j * \langle\left(2 * DICconc_j\right) - ALKconc_j\rangle * \left(1 - \langle 4 * kcarb_j\rangle\right)\right]\right\}^{0.5}$	(51)
Dissolved bicarbonate	$HCO_{3j} = \frac{bicarb_{num}}{1 - \left(4 * kcarb_j\right)}$	(52)
Dissolved carbonate	$CO_{3j} = \frac{ALKconc_j - HCO_{3j}}{2}$	(53)
Dissolved pCO2	$pCO_{2j} = kCO_{2j} * \frac{{HCO_{3j}}^2}{CO_{3j}}$	(54)
Acidity	$Acid_j = kacid * \frac{HCO_{3j}}{CO_{3j}}$	(55)
pH	$pH_j = -1 * \log_{10} Acid_j$	(56)
[Mg]/[Ca] in the ocean during the model run	$\frac{Mg}{Ca}_{MECO} = \frac{Mgconc_s}{Caconc_s}$	(57)
Low latitude surface ocean carbonate solubility rate constant	$solcon_s = 0.7 * \left\{1 - solalpha * \left(\frac{[Mg]}{[Ca]}_0 - \frac{[Mg]}{[Ca]}_{MECO}\right)\right\}$	(58)
High latitude surface ocean carbonate solubility rate constant	$solcon_h = 0.8 * \left\{1 - solalpha * \left(\frac{[Mg]}{[Ca]}_0 - \frac{[Mg]}{[Ca]}_{MECO}\right)\right\}$	(59)
Deep ocean carbonate solubility rate constant	$solcon_d = 1.15 * \left\{1 - solalpha * \left(\frac{[Mg]}{[Ca]}_0 - \frac{[Mg]}{[Ca]}_{MECO}\right)\right\}$	(60)
Calcite saturation	$Saturation_j = \frac{CO_{3j} * Caconc_j}{solcon_j}$	(61)
Saturation coefficient	$sat\_minus\_1 = \max\left(Saturation_s - 1, 0\right)$	(62)
Lysocline depth	$lydp = 5.8 + 50 * \left(CO_{3\,deep} - 0.1\right)$	(63)
Fractional area above the lysocline	$froa = 0.1 + \left(\frac{lydp}{6}\right)^{2.5}$	(64)
Total marine carbonate burial	$Fbc_{total} = sat\_minus\_1^{1.585} * \frac{1}{Saturation_0} * Fbc_0$	(65)
Marine carbonate burial in shelf environments	$Fbc_{shelf} = \frac{1 \cdot 10^{13} * CO_{3s}}{shelf\ fraction}$	(66)

Deep ocean carbonate burial	$Fbc_{deep} = Fbc_{total} - Fbc_{shelf}$	(67)
Terrestrial organic burial	$Fbg_{land} = Fbg_{land\_0}$	(68)
Marine organic burial	$Fbg_{sea} = Fbg_{sea\_0} * bg_{scale}$	(69)
Low latitude surface ocean air-sea C gas exchange	$airsea_s = surf_{area} * A_0 * \frac{1}{tau} * (RCO_2 - pCO_{2s})$	(70)
High latitude surface ocean air-sea C gas exchange	$airsea_h = high_{area} * A_0 * \frac{1}{tau} * (RCO_2 - pCO_{2h})$	(71)
Hydrothermal input of lithium	$Fhyd_{Li} = fG * Fhyd_{Li\_0}$	(72)
Marine authigenic clay sink of lithium	$Fmaac_{Li} = \frac{Liconc_s}{Liconc_{s\_0}} * \left[\left(1 - Li_{split}\right) * Fsink_{Li_0}\right] * \frac{Frw}{Frw_0}$	(73)
Altered oceanic crust sink of lithium	$Faoc_{Li} = \frac{Liconc_d}{Liconc_{d\_0}} * \left(Li_{split} * Fsink_{Li_0}\right) * \frac{Fsfw}{Fsfw_0}$	(74)
Convert silicate weathering from moles per year to tonnes per year	$Fws_{tonnes} = \frac{5.5 \cdot 10^8}{8.7 \cdot 10^{12}} * Fws$	(75)
Convert erosion to tonnes per year	$erosion_{tonnes} = Fws_{tonnes} * fR * E{:}W$	(76)
Weathering intensity	$WI = \frac{Fws_{tonnes}}{Fws_{tonnes} + erosion_{tonnes}}$	(77)
δ^7Li of rivers	$\delta^7 Li_{riv} = \delta^7 Li_{rock} - \left[\left(\frac{1 - WI}{WI}\right) * \left(-a_{li} * e^{\frac{-b_{li}}{WI}}\right)\right] + \left(clayfrac * atemp_{change}\right)$	(78)
Fraction of lithium partitioned from the bedrock into the dissolved load via a Rayleigh distillation function	$ray\_frac = e^{\frac{\delta^7 Li_{riv} - \delta^7 Li_{rock}}{\delta^7 Li_{sec}}}$	(79)
Riverine input of lithium	$Friv_{Li} = Friv_{Li\_0} * \frac{Fws}{Fws_0} * [1 + (ray\_frac - ray\_frac_0)]$	(80)
δ^7Li of the marine authigenic clay sink	$\delta^7 Li_{maac} = \delta^7 Li_s - \left[\Delta^7 Li_{maac} + \left(clayfrac * stemp_{change}\right)\right]$	(81)
δ^7Li of the altered oceanic crust sink	$\delta^7 Li_{aoc} = \delta^7 Li_d - \left[\Delta^7 Li_{aoc} + \left(clayfrac * dtemp_{change}\right)\right]$	(82)
Osmium input from weathering of silicates	$Fws_{Os} = Fws_{Os\_0} * Os_{split} * \frac{Fws}{Fws_0}$	(83)
Osmium input from weathering of organic rich lithologies	$Fwg_{Os} = Fwg_{Os\_0} * \left(1 - Os_{split}\right) * \frac{Fwg}{Fwg_0}$	(84)

Osmium input from low temperature hydrothermal systems	$Flth_{Os} = Flth_{Os\_0} * fG$	(85)
Osmium input from high temperature hydrothermal systems	$Fhth_{Os} = Fhth_{Os\_0} * fG$	(86)
Osmium input to the low latitude surface ocean from aeolian dust	$Fadusts_{Os} = Fadust_{Os\_0} * surf_{area}$	(87)
Osmium input to the high latitude surface ocean from aeolian dust	$Fadusth_{Os} = Fadust_{Os\_0} * high_{area}$	(88)
Osmium input to the low latitude surface ocean from cosmic dust	$Fcdusts_{Os} = Fcdust_{Os\_0} * surf_{area}$	(89)
Osmium input to the high latitude surface ocean from cosmic dust	$Fcdusth_{Os} = Fcdust_{Os\_0} * high_{area}$	(90)
Combined sinks of osmium	$Fsink_{Os} = \frac{Osconc_s}{Osconc_{s\_0}} * Fsink_{Os\_0}$	(91)
Hydrothermal input of calcium	$Fhyd_{Ca} = Fhyd_{Ca\_0} * fG$	(92)
Silicon input to the low latitude surface ocean from aeolian dust	$Fadusts_{Si} = Fadust_{Si\_0} * surf_{area}$	(93)
Silicon input to the high latitude surface ocean from aeolian dust	$Fadusth_{Si} = Fadust_{Si\_0} * high_{area}$	(94)
Hydrothermal input of silicon	$Fhyd_{Si} = Fhyd_{Si\_0} * fG$	(95)
Biogenic sink of silicon	$Fopal_{Si} = \frac{Siconc_s}{Siconc_{s\_0}} * Fopal_{Si\_0}$	(96)
Reverse weathering sink of silicon	$Frw_{Si} = \frac{Siconc_s}{Siconc_{s\_0}} * Frw_{Si_0} * rw_{scale}$	(97)

Note: X represents a constituent (e.g. DIC, Li etc.) and j represents an ocean box (e.g. deep ocean etc.)

Table S15 | Reservoir equations.

Atmospheric CO_2	$\frac{dCO_2}{dt} = Fmg + Fmc + Fwg - Fbg_{land} - Fwc - (2 * Fws) - (2 * Fsfw) - airsea_s - airsea_h + CO_2input$	(98)
Low latitude surface ocean DIC	$\frac{dDIC_s}{dt} = airsea_s + DIC_{d2s} + (2 * Fwc) + (2 * Fws) - Fbg_{sea} - Fbc_{shelf} - DIC_{s2h}$	(99)
High latitude surface ocean DIC	$\frac{dDIC_h}{dt} = airsea_h + DIC_{s2h} - DIC_{h2d}$	(100)
Deep ocean DIC	$\frac{dDIC_d}{dt} = DIC_{h2d} + (2 * Fsfw) - Fbc_{deep} - DIC_{d2s}$	(101)
Low latitude surface ocean alkalinity	$\frac{dALK_s}{dt} = (2 * Fwc) + (2 * Fws) + ALK_{d2s} - (2 * Fbc_{shelf}) - (2 * Frw_{Si}) - ALK_{s2h}$	(102)
High latitude surface ocean alkalinity	$\frac{dALK_h}{dt} = ALK_{s2h} - ALK_{h2d}$	(103)
Deep ocean alkalinity	$\frac{dALK_d}{dt} = ALK_{h2d} + (2 * Fsfw) - (2 * Fbc_{deep}) - ALK_{d2s}$	(104)
$\delta^{13}C$ of atmospheric CO_2 mass balance	$\frac{d\delta^{13}C_a * CO_2}{dt} = (Fmg * \delta^{13}C_g) + (Fmc * \delta^{13}C_c) + (Fwg * \delta^{13}C_g) - (Fbg_{land} * (\delta^{13}C_a - alpha_c)) - (Fwc * \delta^{13}C_a) - (2 * Fws * \delta^{13}C_a) - (2 * Fsfw * \delta^{13}C_{DICs}) - (airsea_s * \delta^{13}C_a) - (airsea_h * \delta^{13}C_a) + (CO_2input * \delta^{13}C_{input})$	(105)
$\delta^{13}C$ of low latitude surface ocean DIC mass balance	$\frac{d\delta^{13}C_{DICs} * DIC_s}{dt} = (airsea_s * \delta^{13}C_a) + (Fwc * \delta^{13}C_c) + (Fwc * \delta^{13}C_a) + (2 * Fws * \delta^{13}C_a) + (DIC_{d2s} * \delta^{13}C_{DICd}) - (DIC_{s2h} * \delta^{13}C_{DICs}) - (Fbg_{sea} * (\delta^{13}C_{DICs} - alpha_c)) - (Fbc_{shelf} * \delta^{13}C_{DICs})$	(106)
$\delta^{13}C$ of high latitude surface ocean DIC mass balance	$\frac{d\delta^{13}C_{DICh} * DIC_h}{dt} = (airsea_h * \delta^{13}C_a) + (DIC_{s2h} * \delta^{13}C_{DICs}) - (DIC_{h2d} * \delta^{13}C_{DICh})$	(107)
$\delta^{13}C$ of deep ocean DIC mass balance	$\frac{d\delta^{13}C_{DICd} * DIC_d}{dt} = (DIC_{h2d} * \delta^{13}C_{DICh}) + (2 * Fsfw * \delta^{13}C_{DICd}) - (Fbc_{deep} * \delta^{13}C_{DICd}) - (DIC_{d2s} * \delta^{13}C_{DICd})$	(108)

Low latitude surface ocean lithium	$$\frac{dLi_s}{dt} = Friv_{Li} + Fd2s_{Li} - Fmaac_{Li} - Fs2h_{Li}$$	(109)
High latitude surface ocean lithium	$$\frac{dLi_h}{dt} = Fs2h_{Li} - Fh2d_{Li}$$	(110)
Deep ocean lithium	$$\frac{dLi_d}{dt} = Fh2d_{Li} + Fhyd_{Li} - Faoc_{Li} - Fd2s_{Li}$$	(111)
δ7Li of low latitude surface ocean mass balance**	$$\frac{d\delta^7Li_s * Li_s}{dt} = (Friv_{Li} * \delta^7Li_{riv}) + (Fd2s_{Li} * \delta^7Li_d) - (Fmaac_{Li} * \delta^7Li_{maac}) - (Fs2h_{Li} * \delta^7Li_s)$$	(112)
δ7Li of high latitude surface ocean mass balance	$$\frac{d\delta^7Li_h * Li_h}{dt} = (Fs2h_{Li} * \delta^7Li_s) - (Fh2d_{Li} * \delta^7Li_h)$$	(113)
δ7Li of deep ocean mass balance**	$$\frac{d\delta^7Li_d * Li_d}{dt} = (Fh2d_{Li} * \delta^7Li_h) + \left(Fhyd_{Li} * \delta^7Li_{hyd}\right) - (Faoc_{Li} * \delta^7Li_{aoc}) - (Fd2s_{Li} * \delta^7Li_d)$$	(114)
Low latitude surface ocean osmium	$$\frac{dOs_s}{dt} = Fws_{Os} + Fwg_{Os} + Flth_{Os} + Fhth_{Os} + Fadusts_{Os} + Fcdusts_{Os} + Fman_{Os} + Fd2s_{Os} - Fsink_{Os} - Fs2h_{Os}$$	(115)
High latitude surface ocean osmium	$$\frac{dOs_h}{dt} = Fadusth_{Os} + Fcdusth_{Os} + Fs2h_{Os} - Fh2d_{Os}$$	(116)
Deep ocean osmium	$$\frac{dOs_d}{dt} = Fh2d_{Os} - Fd2s_{Os}$$	(117)
$^{187}Os/^{188}Os$ of low latitude surface ocean mass balance	$$\frac{d\delta Os_s * Os_s}{dt} = (Fws_{Os} * \delta Os_{ws}) + \left(Fwg_{Os} * \delta Os_{wg}\right) + (Flth_{Os} * \delta Os_{lth}) + (Fhth_{Os} * \delta Os_{hth}) + (Fadusts_{Os} * \delta Os_{adust}) + (Fcdusts_{Os} * \delta Os_{cdust}) + (Fman_{Os} * \delta Os_{man}) + (Fd2s_{Os} * \delta Os_d) - (Fsink_{Os} * \delta Os_s) - (Fs2h_{Os} * \delta Os_s)$$	(118)
$^{187}Os/^{188}Os$ of high latitude surface ocean mass balance	$$\frac{d\delta Os_h * Os_h}{dt} = (Fadusth_{Os} * \delta Os_{adust}) + (Fcdusth_{Os} * \delta Os_{cdust}) + (Fs2h_{Os} * \delta Os_s) - (Fh2d_{Os} * \delta Os_h)$$	(119)
$^{187}Os/^{188}Os$ of deep ocean mass balance	$$\frac{d\delta Os_d * Os_d}{dt} = (Fh2d_{Os} * \delta Os_h) - (Fd2s_{Os} * \delta Os_d)$$	(120)
Low latitude surface ocean calcium	$$\frac{dCa_s}{dt} = Fwc + Fws + Fd2s_{Ca} - Fbc_{shelf} - Fs2h_{Ca} - (0.2 * Frw_{Si})$$	(121)
High latitude surface ocean calcium	$$\frac{dCa_h}{dt} = Fs2h_{Ca} - Fh2d_{Ca}$$	(122)
Deep ocean calcium	$$\frac{dCa_d}{dt} = Fh2d_{Ca} + Fhyd_{Ca} - Fbc_{deep} - Fd2s_{Ca}$$	(123)
Low latitude surface ocean silicon	$$\frac{dSi_s}{dt} = Fadusts_{Si} + Fws + Fd2s_{Si} - Frw_{Si} - Fopal - Fs2h_{Si}$$	(124)

High latitude surface ocean silicon	$\frac{dSi_h}{dt} = Fadusts_{Si} + Fs2h_{Si} - Fh2d_{Si}$	(125)
Deep ocean silicon	$\frac{dSi_d}{dt} = Fh2d_{Si} + Fhyd_{Si} + Fsfw - Fd2s_{Si}$	(126)

** Note that these sink isotope terms include the relevant seawater values and the isotopic fractionation due to clay formation/alteration of oceanic crust – see equations (81) and (82).

References

1. Shipboard Scientific Party. Site 959. in *Proceedings of the Ocean Drilling Program, Initial Reports, Volume 159* (eds. Mascle, J., Lohmann, G. P., Clift, P. D. & Al., E.) (1996). doi:doi: 10.2973/odp.proc.ir.159.105.1996.

2. Cramwinckel, M. J. *et al.* Synchronous tropical and polar temperature evolution in the Eocene letter. *Nature* **559**, 382–386 (2018).

3. Meckler, A. N. *et al.* Cenozoic evolution of deep ocean temperature from clumped isotope thermometry. *Science (80-.).* **377**, 86–90 (2022).

4. Marchitto, T. M. *et al.* Improved oxygen isotope temperature calibrations for cosmopolitan benthic foraminifera. *Geochim. Cosmochim. Acta* **130**, 1–11 (2014).

5. Pogge von Strandmann, P. A. E. *et al.* Assessing bulk carbonates as archives for seawater Li isotope ratios. *Chem. Geol.* **530**, 119338 (2019).

6. van der Ploeg, R. *et al.* Middle Eocene greenhouse warming facilitated by diminished weathering feedback. *Nat. Commun.* **9**, 1–10 (2018).

7. Henehan, M. J. *et al.* Revisiting the Middle Eocene Climatic Optimum "Carbon Cycle Conundrum" With New Estimates of Atmospheric pCO_2 From Boron Isotopes. *Paleoceanogr. Paleoclimatology* **35**, (2020).

8. Foster, G. L., Royer, D. L. & Lunt, D. J. Future climate forcing potentially without precedent in the last 420 million years. *Nat. Commun.* **8**, 1–8 (2017).

9. *The Geologic Time Scale 2012*. (Elsevier B.V., 2012).

10. *The Geologic Time Scale 2020*. (Elsevier B.V., 2020).

11. Hansen, J., Sato, M., Russell, G. & Kharecha, P. Climate sensitivity, sea level and atmospheric carbon dioxide. *Philos. Trans. R. Soc. A Math. Phys. Eng. Sci.* **371**, (2013).

12. Pracht, H., Metcalfe, B. & Peeters, F. J. C. Oxygen isotope composition of the final chamber of planktic foraminifera provides evidence of vertical migration and depth-integrated growth. *Biogeosciences* **16**, 643–661 (2019).

13. Berner, R. A. GEOCARBSULF: A combined model for Phanerozoic atmospheric O_2 and CO_2. *Geochim. Cosmochim. Acta* **70**, 5653–5664 (2006).

14. Lenton, T. M., Daines, S. J. & Mills, B. J. W. COPSE reloaded: An improved model of biogeochemical cycling over Phanerozoic time. *Earth-Science Rev.* **178**, 1–28 (2018).

15. Krause, A. J. *et al.* Stepwise oxygenation of the Paleozoic atmosphere. *Nat. Commun.* **9**, 1–10 (2018).

16. Pogge von Strandmann, P. A. E., Jenkyns, H. C. & Woodfine, R. G. Lithium isotope evidence for enhanced weathering during Oceanic Anoxic Event 2. *Nat. Geosci.* **6**, 668–672 (2013).

17. Wong, K. *et al.* Deep Carbon Cycling Over the Past 200 Million Years: A Review of Fluxes in Different Tectonic Settings. *Front. Earth Sci.* **7**, 1–22 (2019).

18. Guo, Z., Wilson, M., Dingwell, D. B. & Liu, J. India-Asia collision as a driver of atmospheric CO_2 in the Cenozoic. *Nat. Commun.* **12**, 1–15 (2021).

19. van der Boon, A. *et al.* Exploring a link between the Middle Eocene Climatic Optimum and Neotethys continental arc flare-up. *Clim. Past* **17**, 229–239 (2021).

20. Berner, R. A. Inclusion of the weathering of volcanic rocks in the GEOCARBSULF model. *Am. J. Sci.* **306**, 295–302 (2006).

21. Brune, S., Williams, S. E. & Müller, R. D. Potential links between continental rifting, CO_2 degassing and climate change through time. *Nat. Geosci.* **10**, (2017).

22. Royer, D. L., Donnadieu, Y., Park, J., Kowalczyk, J. & Goddéris, Y. Error analysis of CO_2 and O_2 estimates from the long-term geochemical model GEOCARBSULF. *Am. J. Sci.* **314**, 1259–1283 (2014).

23. Krause, A. J., Mills, B. J. W., Merdith, A. S., Lenton, T. M. & Poulton, S. W. Extreme variability in atmospheric oxygen levels in the late Precambrian. *Sci. Adv.* **8**, eabm8191 (2022).

24. Van Der Boon, A. *et al.* Onset of Maikop sedimentation and cessation of Eocene arc volcanism in the Talysh Mountains, Azerbaijan. *Geol. Soc. Spec. Publ.* **428**, 145–169 (2017).

25. Keskin, M., Genç, Ş. C. & Tüysüz, O. Petrology and geochemistry of post-collisional Middle Eocene volcanic units in North-Central Turkey: Evidence for magma generation by slab breakoff following the closure of the Northern Neotethys Ocean. *Lithos* **104**, 267–305 (2008).

26. Johansson, L., Zahirovic, S. & Müller, R. D. The interplay between the eruption and weathering of Large Igneous Provinces and the deep-time carbon cycle. *Geophys. Res. Lett.* **45**, 5380–5389 (2018).

27. Rooney, T. O. The Cenozoic magmatism of East-Africa: Part I — Flood basalts and pulsed magmatism. *Lithos* **286**–**287**, 264–301 (2017).

28. George, R., Rogers, N. & Kelley, S. Earliest magmatism in Ethiopia: Evidence for two mantle plumes in one flood basalt province. *Geology* **26**, 923–926 (1998).

29. Hay, W. W. *et al.* Evaporites and the salinity of the ocean during the Phanerozoic: Implications for climate, ocean circulation and life. *Palaeogeogr. Palaeoclimatol. Palaeoecol.* **240**, 3–46 (2006).

30. Mills, B. J. W. *et al.* Modelling the long-term carbon cycle, atmospheric CO_2, and Earth surface temperature from late Neoproterozoic to present day. *Gondwana Res.* **67**, 172–186 (2019).

31. Rodríguez-Molina, S., González, P. J., Charco, M., Negredo, A. M. & Schmidt, D. A. Time-

Scales of Inter-Eruptive Volcano Uplift Signals: Three Sisters Volcanic Center, Oregon (United States). *Front. Earth Sci.* **8**, 1–19 (2021).

32. Siewert, J. & Ferlito, C. Mechanical erosion by flowing lava. *Contemp. Phys.* **49**, 43–54 (2008).

33. Pruski, F. F. & Nearing, M. A. Climate-induced changes in erosion during the 21st century for eight U.S. locations. *Water Resour. Res.* **38**, 34-1-34–11 (2002).

34. Nearing, M. A. *et al.* Modeling response of soil erosion and runoff to changes in precipitation and cover. *Catena* **61**, 131–154 (2005).

35. Spicer, R. A. *et al.* Why 'the uplift of the Tibetan Plateau' is a myth. *Natl. Sci. Rev.* **8**, (2021).

36. Gale, A. S., Jeffery, P. A., Huggett, J. M. & Connolly, P. Eocene inversion history of the Sandown Pericline, Isle of Wight, southern England. *J. Geol. Soc. London.* **156**, 327–339 (1999).

37. Scotese, C. R. PALEOMAP: PaleoAtlas for GPlates and the PaleoData Plotter Program. (2016).

38. Berner, R. A. & Kothavala, Z. GEOCARB III; a revised model of atmospheric CO_2 over Phanerozoic time. *Am. J. Sci.* **301**, 182–204 (2001).

39. Larsen, I. J., Montgomery, D. R. & Greenberg, H. M. The contribution of mountains to global denudation. *Geology* **42**, 527–530 (2014).

40. Milliman, J. & Farnsworth, K. *River Discharge to the Coastal Ocean: A Global Synthesis*. (Cambridge University Press, 2011).

41. Milliman, J. D. & Syvitski, J. P. M. Geomorphic/Tectonic Control of Sediment Discharge to the Ocean: The Importance of Small Mountainous Rivers. *J. Geol.* **100**, 525–544 (1992).

42. Caves Rugenstein, J. K., Ibarra, D. E. & von Blanckenburg, F. Neogene cooling driven by land surface reactivity rather than increased weathering fluxes. *Nature* **571**, 99–102 (2019).

43. Tipper, E. T. *et al.* Global silicate weathering flux overestimated because of sediment-water cation exchange. *Proc. Natl. Acad. Sci. U. S. A.* **118**, (2020).

44. Gaillardet, J., Dupré, B., Louvat, P. & Allègre, C. J. Global silicate weathering and CO_2 consumption rates deduced from the chemistry of large rivers. *Chem. Geol.* **159**, 3–30 (1999).

45. Bouchez, J., Von Blanckenburg, F. & Schuessler, J. A. Modeling novel stable isotope ratios in the weathering zone. *Am. J. Sci.* **313**, 267–308 (2013).

46. Dellinger, M. *et al.* Riverine Li isotope fractionation in the Amazon River basin controlled by the weathering regimes. *Geochim. Cosmochim. Acta* **164**, 71–93 (2015).

47. Steinhoefel, G., Brantley, S. L. & Fantle, M. S. Lithium isotopic fractionation during weathering and erosion of shale. *Geochim. Cosmochim. Acta* **295**, 155–177 (2021).

48. Pogge von Strandmann, P. A. E., Kasemann, S. A. & Wimpenny, J. B. Lithium and Lithium Isotopes in Earth's Surface Cycles. *Elements* **16**, 253–258 (2020).

49. Sluijs, A., Zeebe, R. E., Bijl, P. K. & Bohaty, S. M. A middle Eocene carbon cycle conundrum. *Nat. Geosci.* **6**, 429–434 (2013).

50. Rae, J. W. B. *et al.* Atmospheric CO_2 over the past 66 million years from marine archives. *Annu. Rev. Earth Planet. Sci.* **49**, 609–641 (2021).

51. Ernst, R. E. *et al.* Large Igneous Province Record Through Time and Implications for Secular Environmental Changes and Geological Time-Scale Boundaries. in *Large Igneous Provinces: A Driver of Global Environmental and Biotic Changes* (eds. Ernst, R. E., Dickson, A. J. & Bekker, A.) 1–26 (2021). doi:10.1002/9781119507444.ch1.

52. Misra, S. & Froelich, P. N. Lithium isotope history of cenozoic seawater: Changes in silicate weathering and reverse weathering. *Science (80-.).* **335**, 818–823 (2012).

53. Vigier, N. & Goddéris, Y. A new approach for modeling Cenozoic oceanic lithium isotope paleo-variations: The key role of climate. *Clim. Past* **11**, 635–645 (2015).

54. Gaskell, D. E. *et al.* The latitudinal temperature gradient and its climate dependence as inferred from foraminiferal $\delta^{18}O$ over the past 95 million years. *Proc. Natl. Acad. Sci. U. S. A.* **119**, 1–8 (2022).

55. Scotese, C. R., Song, H., Mills, B. J. W. & van der Meer, D. G. Phanerozoic paleotemperatures: The earth's changing climate during the last 540 million years. *Earth-Science Rev.* **215**, 103503 (2021).

56. Zachos, J., Pagani, H., Sloan, L., Thomas, E. & Billups, K. Trends, rhythms, and aberrations in global climate 65 Ma to present. *Science (80-.).* **292**, 686–693 (2001).

57. van der Ploeg, R. *et al.* North Atlantic surface ocean warming and salinization in response to middle Eocene greenhouse warming. *Sci. Adv.* **9**, 1–16 (2023).

58. Dixon, J. L. & von Blanckenburg, F. Soils as pacemakers and limiters of global silicate weathering. *Comptes Rendus - Geosci.* **344**, 597–609 (2012).

59. Ito, A. & Wagai, R. Data Descriptor: Global distribution of clay-size minerals on land surface for biogeochemical and climatological studies. *Sci. Data* **4**, 1–11 (2017).

60. Tardy, Y., Krempp, G. & Trauth, N. Le lithium dans les minéraux argileux des ciments et des sols. *Geochemica Cosmochim. Acta* **36**, 397–412 (1972).

61. Westerhold, T. & Röhl, U. Orbital pacing of Eocene climate during the Middle Eocene Climate Optimum and the chron C19r event: Missing link found in the tropical western Atlantic. *Geochemistry, Geophys. Geosystems* **14**, 4811–4825 (2013).

62. Giorgioni, M. *et al.* Carbon cycle instability and orbital forcing during the Middle Eocene Climatic Optimum. *Sci. Rep.* **9**, 1–10 (2019).

63. Lu, X., Kendall, B., Stein, H. J. & Hannah, J. L. Temporal record of osmium concentrations and 187Os/188Os in organic-rich mudrocks: Implications for the osmium geochemical cycle and the use of osmium as a paleoceanographic tracer. *Geochim. Cosmochim. Acta* **216**, 221–241 (2017).

64. Masaitis, V. L. Impact structures of northeastern Eurasia : The territories of Russia and adjacent countries. *Meteorit. Planet. Sci.* **34**, 691–711 (1999).

65. Pogge von Strandmann, P. A. E. *et al.* Variations of Li and Mg isotope ratios in bulk chondrites and mantle xenoliths. *Geochim. Cosmochim. Acta* **75**, 5247–5268 (2011).

66. Dal Corso, J. *et al.* Permo–Triassic boundary carbon and mercury cycling linked to terrestrial ecosystem collapse. *Nat. Commun.* **11**, 1–9 (2020).

67. Berner, R. A. A model for Atmospheric CO_2 over Phanerozoic Time. *Am. J. Sci.* **291**, 339–376 (1991).

68. Berner, R. A. 3GEOCARBII: A Revised Model of Atmospheric CO_2 over Phanerozoic Time. *Am. J. Sci.* **294**, 56–91 (1994).

69. Zeebe, R. E. LOSCAR: Long-term Ocean-atmosphere-Sediment CArbon cycle Reservoir model v2.0.4. *Geosci. Model Dev.* **5**, 149–166 (2012).

70. Lowenstein, T. K., Kendall, B. & Anbar, A. D. *The Geologic History of Seawater*. *Treatise on Geochemistry: Second Edition* vol. 8 (Elsevier Ltd., 2013).

71. Horita, J., Zimmermann, H. & Holland, H. D. Chemical evolution of seawater during the Phanerozoic. *Geochim. Cosmochim. Acta* **66**, 3733–3756 (2002).

72. Kalderon-Asael, B. *et al.* A lithium-isotope perspective on the evolution of carbon and silicon cycles. *Nature* **595**, 6–11 (2021).

73. Georg, R. B., West, A. J., Vance, D., Newman, K. & Halliday, A. N. Is the marine osmium isotope record a probe for CO_2 release from sedimentary rocks? *Earth Planet. Sci. Lett.* **367**, 28–38 (2013).

74. Mills, B. J. W., Daines, S. J. & Lenton, T. M. Changing tectonic controls on the long-term carbon cycle from Mesozoic to present. *Geochemistry Geophys. Geosystems* **15**, 4866–4884 (2014).

75. Farkaš, J. *et al.* Calcium isotope record of Phanerozoic oceans: Implications for chemical evolution of seawater and its causative mechanisms. *Geochim. Cosmochim. Acta* **71**, 5117–5134 (2007).

76. Tréguer, P. J. & De La Rocha, C. L. The world ocean silica cycle. *Ann. Rev. Mar. Sci.* **5**, 477–501 (2013).

77. Rahman, S., Tamborski, J. J., Charette, M. A. & Cochran, J. K. Dissolved silica in the subterranean estuary and the impact of submarine groundwater discharge on the global marine silica budget. *Mar. Chem.* **208**, 29–42 (2019).

78. Liu, C. *et al.* Lithium Extraction from Seawater through Pulsed Electrochemical Intercalation. *Joule* **4**, 1459–1469 (2020).
